# Klotho prevents transforming growth factor-β2-induced senescent-like morphological changes in the retinal pigment epithelium

**DOI:** 10.1038/s41419-023-05851-8

**Published:** 2023-05-20

**Authors:** Ha Young Jang, Soo-Jin Kim, Kyu-Sang Park, Jeong Hun Kim

**Affiliations:** 1 Fight against Angiogenesis-Related Blindness (FARB) Laboratory, Clinical Research Institute, Seoul National University Hospital, Seoul, Republic of Korea; 2 Department of Physiology, Yonsei University Wonju College of Medicine, Wonju, Republic of Korea; 3 Mitohormesis Research Center, Yonsei University Wonju College of Medicine, Wonju, Republic of Korea; 4 Department of Ophthalmology, College of Medicine, Seoul National University, Seoul, Republic of Korea; 5 Institute of Reproductive Medicine and Population, Seoul National University College of Medicine, Seoul, Republic of Korea

**Keywords:** Mechanisms of disease, Macular degeneration

## Abstract

Degenerative changes of the retinal pigment epithelium (RPE) triggered by transforming growth factor-β2 (TGF-β2) and oxidative stress play a critical role in the progression of age-related macular degeneration (AMD). The expression of α-klotho, an antiaging protein, declines with age, increasing the risk factors for age-related diseases. Here, we investigated the protective effects of soluble α-klotho on TGF-β2-induced RPE degeneration. The morphological changes induced by TGF-β2, including epithelial-mesenchymal transition (EMT), were attenuated in the mouse RPE by the intravitreal injection (IVT) of α-klotho. In ARPE19 cells, EMT and morphological alterations by TGF-β2 were attenuated by co-incubation with α-klotho. TGF-β2 decreased miR-200a accompanied by zinc finger e-box binding homeobox1 (ZEB1) upregulation and EMT, all of which were prevented by α-klotho co-treatment. Inhibitor of miR-200a mimicked TGF-β2-induced morphological changes, which were recovered by ZEP1 silencing, but not by α-klotho, implying the upstream regulation of α-klotho on miR-200a-ZEP1-EMT axis. α-Klotho inhibited receptor binding of TGF-β2, Smad2/3 phosphorylation, extracellular signal-regulated protein kinase 1/2 (ERK1/2)-a mechanistic target of rapamycin (mTOR) activation and oxidative stress via NADPH oxidase 4 (NOX4) upregulation. Furthermore, α-klotho recovered the TGF-β2-induced mitochondrial activation and superoxide generation. Interestingly, TGF-β2 upregulated α-klotho expression in the RPE cells, and genetic suppression of endogenous α-klotho aggravated TGF-β2-induced oxidative stress and EMT. Lastly, α-klotho abrogated senescence-associated signaling molecules and phenotypes induced by long-term incubation with TGF-β2. Hence, our findings indicate that the antiaging α-klotho plays a protective role against EMT and degeneration of the RPE, demonstrating the therapeutic potential for age-related retinal diseases, including the dry type of AMD.

## Introduction

The retinal pigment epithelium (RPE) is a hexagon-cuboidal pigmented cell layer between the neuronal retina and choroid [1]. As the RPE performs a number of critical functions to maintain and ensure the survival of overlying photoreceptor cells, loss of RPE characteristics are closely related to the pathogenesis of retinal degenerative disorders, including age-related macular degeneration (AMD). RPE cells degenerate and lose their epithelial integrity to survive in a harsh microenvironment caused by the progression of AMD and migrate to other retinal tissues through a transformation of mesenchymal properties [2, 3]. Epithelial-mesenchymal transition (EMT) is conditionally reversible, which could be an important target for novel therapeutics to alleviate the dry type of AMD [3, 4].

TGF-β is a multifunctional cytokine that plays an essential role in the physiological processes involved in the development and wound repair. In addition, the pathophysiologic actions of TGF-β have been suggested to participate in various ocular diseases, including inflammatory, proliferative, and degenerative diseases [5]. Accordingly, TGF-β suppression was found to inhibit both RPE EMT and senescence and blockage of choroidal neovascularization and subretinal fibrosis in animal models [6]. In mammalian tissues, TGF-β exists in three isoforms, namely, TGF-β1, -β2, and -β3 [7]. TGF-β1, the most extensively studied isoform, participates in neovascular AMD [8]. However, in the neural retina and RPE-choroid complexes, the predominant isoform is TGF-β2 [9, 10]. Indeed, the RPE cells or photoreceptors are not only the source but also the target of TGF-β2, establishing physiologic and pathophysiologic autocrine signaling in retinal tissues [9]. The increased concentration of TGF-β2 in the vitreous is associated with the progression of retinal fibrosis in patients with proliferative vitreoretinopathy, a disorder of post-retinal detachment and retinal fibrosis [7]. As the TGF-β2 levels in the vitreous also correlate with the severity of EMT-related fibrosis in the RPE, TGF-β2 could be involved in AMD pathology [11]. Recently, a negative feedback loop between microRNAs (miR-34 and miR-200a) and SNAIL1/zinc finger e-box binding homeobox 1 (ZEB1) signaling related to TGF-β-induced EMT has been reported. This feedback regulation mediates autocrine TGF-β signaling, which attenuates the inhibitory control of microRNAs on SNAIL1/ZEB1 and participates in EMT and fibrosis in many different cell types [4, 12, 13].

Klotho is a putative aging suppressor gene encoding a single-pass transmembrane protein with three subfamilies, α-, β-, and γ-klotho [14]. In particular, the levels of circulating soluble α-klotho, a cleaved and secreted fraction of α-klotho, continue to decline with aging and the progression of age-related diseases [15]. α-Klotho-deficient mice (*kl*^-/-^) showed short lifespans with degenerated photoreceptors and the RPE structure, aberrant melanosome distribution, and damaged mitochondria [16]. Klotho mRNA and protein are detected in the retina, and the absence of α-klotho results in the slow deterioration of retinal function [17]. In vitro application of soluble α-klotho increased RPE phagocytosis and protected the RPE from oxidative stress [16]. However, the molecular mechanisms underlying the protective effect of klotho against retinal degeneration remain unclear.

Here, we investigated the inhibitory role of α-klotho on TGF-β2-induced senescence-like morphological changes and oxidative stress of the RPE by suppressing miR200a-ZEB1 axis regulation and oxidative stress generation from NADPH oxidase 4 (NOX4) and mitochondrial respiration. These results provide evidence for the physiologic importance and therapeutic potential of α-klotho as an antiaging strategy against age-related degenerative diseases.

## Results

### α-Klotho attenuates age-related degenerative changes without causing toxicity in vivo

**Fig. 1:**
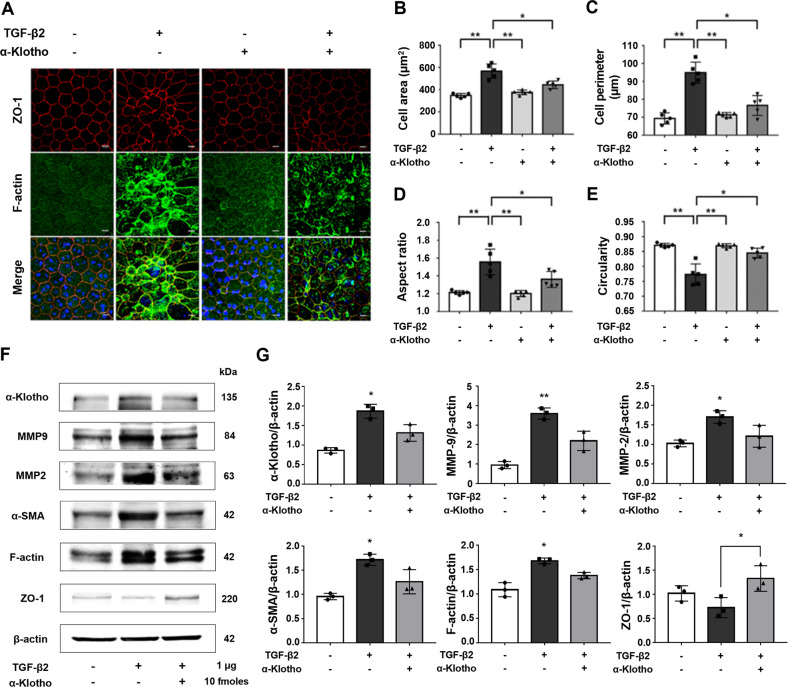
α-Klotho attenuates TGF-β2-induced degenerative changes and degeneration of the RPE in mice. Mice received an IVT injection (2 µL) of vehicle (PBS) or 1 µg of recombinant mouse TGF-β2 with or without 10 fmoles of α-klotho. Three days after the injection, all the mice were sacrificed. **A** Representative immunofluorescence images of the RPE stained for ZO-1 and F-actin (*n* = 5). α-Klotho attenuated the EMT and degenerative changes of the RPE induced by IVT injection of TGF-β2. **B** Morphological analysis of cell size (μm^2^). **C** Morphological analysis of cell perimeter (μm). **D** Morphological analysis of aspect ratio. **E** Morphological analysis of circularity. Scale bar 10 μm. **F** IVT injection of α-klotho suppressed retinal structure remodeling by increasing epithelial markers (ZO-1) and inhibiting mesenchymal markers (α-SMA and F-actin) with MMPs (MMP2 and MMP9) proteins isolated from the mouse RPE (*n* = 5). **G** Quantification of the western blot analysis. Data from three experiments (mean ± SE) are shown in bars and were analyzed by ANOVA. Tukey post hoc test: *p < 0.05. **p < 0.01.

TGF-β2 (1 μg) was intravitreally injected into the mouse eye to establish whether TGF-β2 induces RPE EMT or degenerative changes. The degenerative changes of the RPE due to the breakdown of the outer blood barrier led to an increase in cell size and irregularity, which was evaluated through morphometric analysis, including cell area, perimeter, aspect ratio, and circularity of the RPE cells [18–22]. Co-treatment with α-klotho markedly suppressed the morphological alterations that were significant by TGF-β2 injection, including irregular geometry and stress fiber development (Fig. 1A and Supplementary Fig. S1). α-Klotho significantly restored the increased average cell area (Fig. 1B), perimeter (Fig. 1C), aspect ratio (Fig. 1D) with decreased circularity due to TGF-β2 (Fig. 1E). The IVT injection dose was set to 10 fmoles (10 nM) of α-klotho as there was evidence of an increase in cell size without significance at 30 fmoles (Supplementary Fig. S2). In the western blot assay, α-klotho increased the epithelial marker (ZO-1) and suppressed α-smooth muscle actin (α-SMA), a mesenchymal marker. Furthermore, α-klotho reduced the expression levels of matrix metalloprotease (MMP)-2 and MMP-9 (Fig. 1F and G). As previously reported, α-klotho has endogenously expressed in the mouse RPE tissues [16]. Notably, TGF-β2 application elicited a pronounced upregulation of endogenous α-klotho expression, which was repressed by exogenous α-klotho. Taken together, we can infer that α-klotho plays a protective role against TGF-β2-induced morphological changes similar to the degenerative progress of the RPE.

### α-Klotho inhibits TGF-β2-induced morphological alterations by upregulating miR-200a

**Fig. 2:**
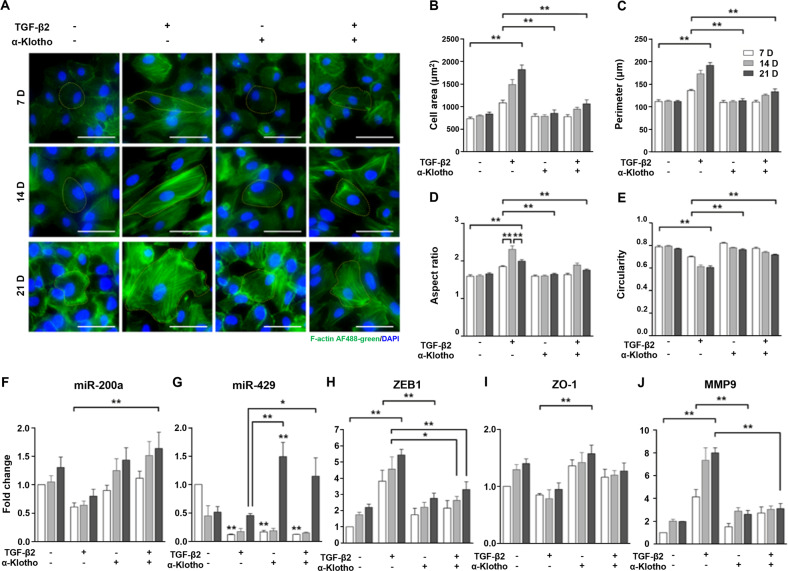
α-Klotho inhibits the TGF-β2-induced morphological alterations by upregulating miR-200a. **A** Representative immunofluorescence images of ARPE19 cells stained for F-actin. ARPE19 cells were exposed to TGF-β2 for 21 days to maximize EMT and cellular senescence, and α-klotho (1 nM) inhibits TGF-β2 induced morphological alterations. **B** Morphological analysis of cell size. **C** Morphological analysis of cell perimeter. **D** Morphological analysis of aspect ratio. **E** Morphological analysis of circularity. Scale bar 100 μm. **F-G** MiR-specific qPCR analysis of the expression of miR-200a (**F**) and -429 (**G**). **H-J** Real-time RT-PCR analysis of the expression of ZEB1 (**H**), ZO-1 (**I**), and MMP-9 (**J**). α-Klotho enhances miR-200a and -429 expression while suppressing ZEB1 expression over 21 days. Data from three experiments (mean ± SE) are shown in bars and were analyzed by ANOVA. Tukey post hoc test: *p < 0.05. **p < 0.01.

To determine the molecular mechanism of α-klotho’s suppressive effects on morphological alterations, we elicited maximized morphological changes in ARPE19 cells following stimulation with TGF-β2 (10 ng/ml) with or without α-klotho (1 nM) over 21 days. ARPE19 cells acquired a spindle-shaped mesenchymal phenotype in response to TGF-β2 over 14 days (Fig. 2A and D). α-Klotho significantly attenuated morphological alterations, such as an extensively increased cell size (Fig. 2B and C), irregularity (Fig. 2E), and stress fiber formation induced following TGF-β2 incubation for 21 days. Co-incubation with α-klotho significantly attenuated senescence-related morphological alterations (Fig. 2A-E). No morphological abnormalities were observed in cells treated with α-klotho alone.

TGF-β2-induced morphological alterations were concomitant with the downregulation of miR-200a (Fig. 2F) and miR-429 (Fig. 2G) and upregulation of ZEB1 (Fig. 2H), which led to a decrease in ZO-1 and elevation of MMP-9. α-Klotho recovered the expression levels of mRNA200a and miR429 from TGF-β2-induced suppression (Fig. 2F and G). As a consequence, the transcriptional level of ZEB1 returned to the basal level by α-klotho, which was followed by an increase in ZO-1 (Fig. 2I) and a decline in MMP-9 (Fig. 2J). These results suggest that the regulation of the miR-200a-ZEB1 axis involves, at least in part, the inhibitory actions of α-klotho against TGF-β2-induced EMT of the RPE. It is worth noting that miR-200a and -429 were well expressed, whereas the other family members of the miR-200 family (miR-200b, -200c, and -141) were barely detectable in ARPE19 cells.

### Senescence-like morphological alterations depend on the miR-200a-ZEB1 axis

**Fig. 3:**
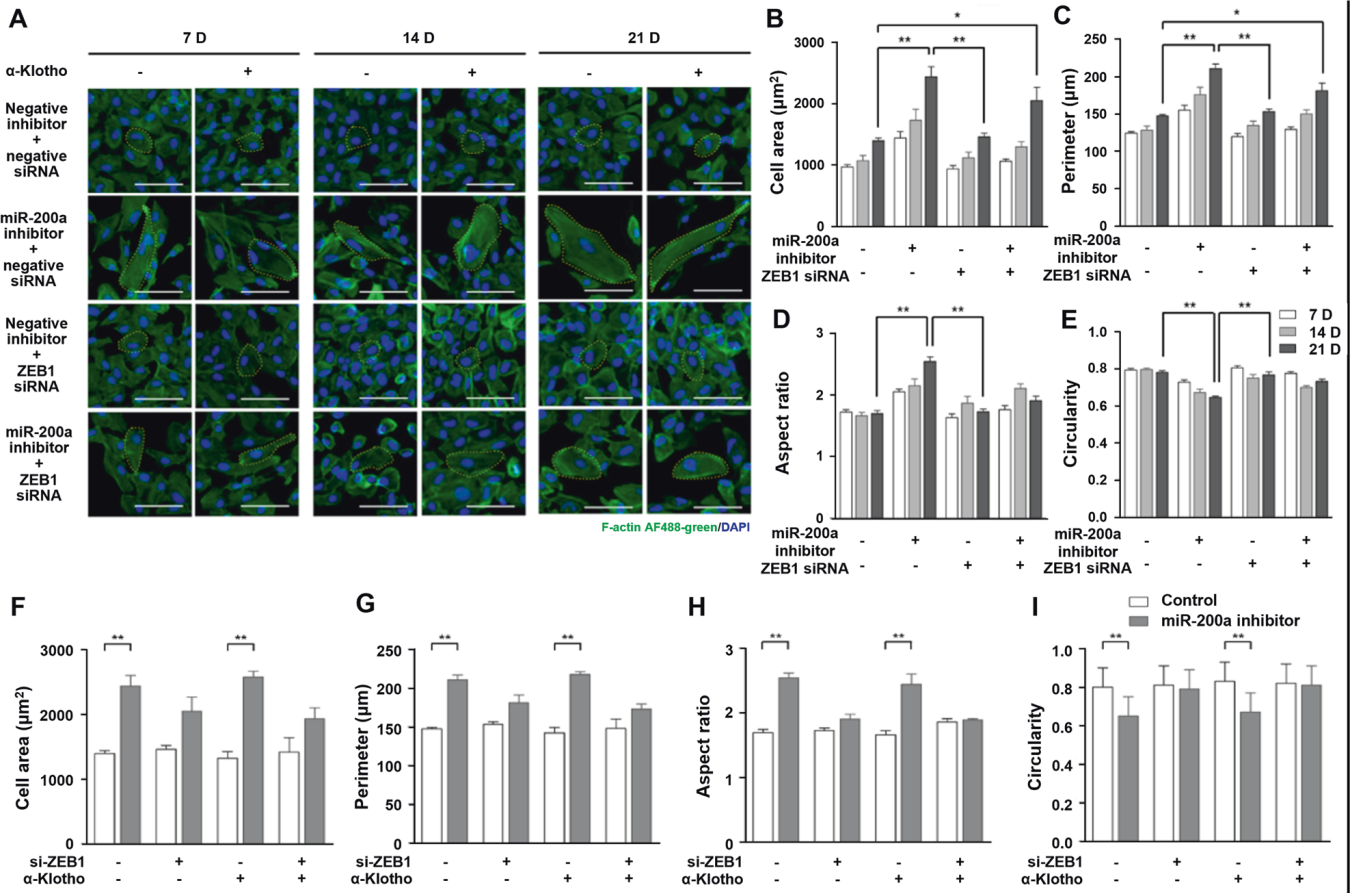
Senescence-like related morphological alterations are regulated by miR-200a-ZEB1 axis. **A** Representative immunofluorescence images of ARPE19 cells transfected with negative control or miR-200a inhibitors or a combination of ZEB1 siRNA for 21 days. Inhibition of miR-200a induces EMT, while ZEB1 inhibition partially suppresses EMT induced by miR-200a inhibition. **B** Morphological analysis of cell size. **C** Morphological analysis of cell perimeter. **D** Morphological analysis of aspect ratio. **E** Morphological analysis of circularity. Scale bar 100 μm. **F-I** There are no significant differences with or without α-klotho in morphological changes analysis including cell size (**F**), perimeter (**G**), aspect ratio (**H**) and circularity (**I**) caused by the downregulated miR-200a and ZEB1 for 21 days. Scale bar 100 μm. Data from three experiments (mean ± SE) are shown in bars and were analyzed by ANOVA. Tukey post hoc test: *p < 0.05. **p < 0.01.

To confirm the role of the miR-200a-ZEB1 axis in morphological phenotypes of ARPE19 cells, we examined the effect of the miR-200a inhibitor and ZEB1 siRNAs. Seven days after transfection with a miR-200a inhibitor, ARPE19 cells had significantly adopted a mesenchymal-like morphology, which intensified over 21 days (Fig. 3A). ZEB1 knockdown was sufficient to prevent EMT initiation by maintaining epithelial integrity. Simultaneous knockdown of ZEB1 attenuated the EMT-related morphological alterations caused by the inhibition of miR-200a, indicating that ZEB1 might be upregulated by miR-200a suppression in RPE cells (Fig. 3A-E). In addition, we observed that α-klotho co-incubation did not affect the morphological changes induced by the inhibition of miR-200 and ZEB1 (Fig. 3F-I), suggesting that α-klotho could be an upstream regulator of the miR-200-ZEB1 axis in the recovery of morphological alterations.

### α-Klotho inhibits TGF-β2-induced EMT by blocking the TGF-β/TGF-βRII interaction

**Fig. 4:**
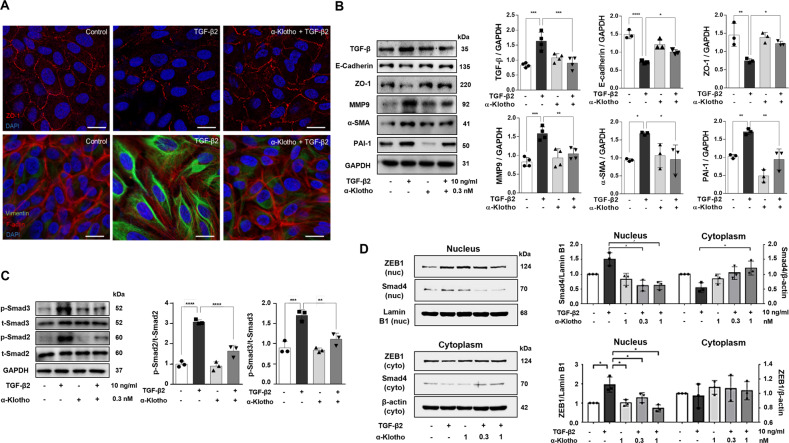
α-Klotho inhibits TGF-β2-induced EMT by blocking the TGF-β/TGF-βRII interaction. **A** Representative immunofluorescence images of ARPE19 cells stained for ZO-1 and vimentin. **B** Immunoblotting analysis showing the expression levels of TGF-β, E-cadherin, ZO-1, MMP-9, α-SMA, and PAI-1. **C, D** α-Klotho treatment suppressed phosphorylation of Smad2 and 3 (**C**) and ZEB1 and Smad4 translocation into the nucleus (**D**). Data from three experiments (mean ± SE) are shown in bars with scatter plots and were analyzed by ANOVA. Tukey post hoc test: *p < 0.05, **p < 0.01, ***p < 0.001 and ****p < 0.0001.

To verify whether α-klotho inhibits TGF-β2-induced EMT and fibrosis, ARPE19 cells were incubated with TGF-β2 (10 ng/ml) in the absence or presence of α-klotho (1 nM). Immunocytochemistry results showed that α-klotho strongly reversed the TGF-β2-induced changes in the EMT markers ZO-1 and vimentin (Fig. 4A). Immunoblotting analysis also demonstrated that α-klotho recovered the TGF-β2-induced EMT by increasing the levels of epithelial markers, such as ZO-1 and E-cadherin, and suppressing those of mesenchymal markers, such as α-SMA, plasminogen activator inhibitor-1 (PAI-1), and MMP-9 (Fig. 4B). Notably, TGF-β2 increased endogenous TGF-β expression, eliciting autocrine or paracrine activation of TGF-β signaling, which is consistent to our previous report [21]. Downstream of the TGF-β receptor, activation of Smad signaling is essential for TGF-β-induced EMT [23]. In ARPE19 cells, TGF-β2-induced phosphorylation of Smad 2 and 3 was abolished by α-klotho co-treatment (Fig. 4C). Furthermore, nuclear translocation of cytoplasmic Smad4, as co-Smad signaling, and ZEB1 was prevented by α-klotho (Fig. 4D), resulting in the repression of EMT and fibrosis.

### α-Klotho inhibits TGF-β2-induced cytosolic and mitochondrial oxidative stress

**Fig. 5:**
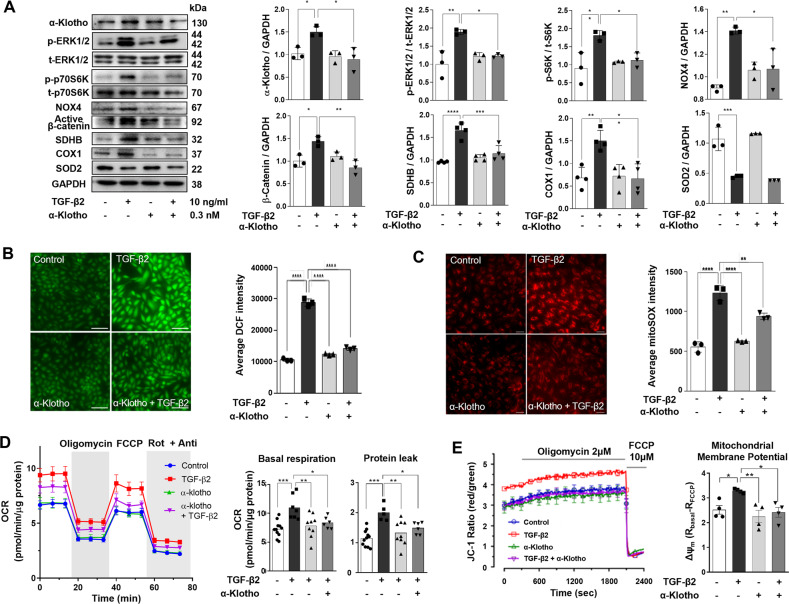
α-Klotho suppresses the TGF-β2-induced cytosolic and mitochondrial oxidative stress. **A** Immunoblotting analysis showing the protein levels of endogenous α-klotho, phosphorylated and total forms of ERK1/2 and p70S6K, as well as NOX4, active catenin, SDHB, COX1, and SOD2. **B** Cytosolic ROS measurement using DCF fluorescence imaging (*n* = 3). **C** Mitochondrial superoxide measurement using mitoSox fluorescence imaging (*n* = 3). For cytosolic ROS and mitochondrial superoxide measurement, each case is the average value of the fluorescence intensities of more than 20 cells on a coverslip. **D** Mitochondrial oxygen consumption measurement and comparison of basal respiration and proton leak (*n* = 9 ~ 11). **E** Mitochondrial membrane potential measurement using JC-1 fluorescence measurement (*n* = 4). Data are presented as the mean ± SE and analyzed by ANOVA. Tukey post hoc test: *p < 0.05, **p < 0.01, ***p < 0.001 and ****p < 0.0001.

Similar to mouse RPE tissues, ARPE19 cells also express endogenous α-klotho, which is further enhanced by TGF-β2 stimulation. Exogenous α-klotho application prevented TGF-β2-induced upregulation of endogenous α-klotho, possibly due to suppression of TGF-β2 signaling (Fig. 5A). We have previously demonstrated that TGF-β1 induces NOX4 upregulation via extracellular signal-regulated protein kinase 1/2 (ERK1/2)-a mechanistic target of rapamycin 1 (mTORC1) activation, which accelerates ROS generation and EMT in the RPE [24]. In this study, we observed that TGF-β2 also initiated phosphorylation of ERK1/2 and p70S6K, downstream of mTORC1 activation, which was prevented by α-klotho co-incubation (Fig. 5A). Notably, α-klotho abolished TGF-β2-induced NOX4 upregulation. Cytosolic ROS measurement using DCF fluorescence imaging demonstrated that α-klotho abrogated the TGF-β2-induced oxidative stress (Fig. 5B). Consistent with a previous report on the positive feedforward loop between NOX4 and the Wnt/β-catenin pathway [25], α-klotho decreased the levels of active β-catenin, which is involved in the EMT of ARPE19 cells (Fig. 5A).

It has been reported that TGF-β increases mitochondrial ROS generation related to mTOR-mediated activation of mitochondrial oxidative phosphorylation [26]. We also observed that mitochondrial superoxide generation was accelerated by TGF-β2, estimated by mitoSox fluorescence imaging experiments (Fig. 5C). TGF-β2 upregulated essential components of mitochondrial electron transport chain (ETC) proteins such as succinate dehydrogenase subunit B (SDHB) and cytochrome c oxidase 1 (COX1) (Fig. 5A). These changes are associated with increased basal respiration and proton leak after TGF-β2 treatment in mitochondrial oxygen consumption measurements (Fig. 5D). The hyperpolarization of the mitochondrial membrane potential (ΔΨ_m_) by TGF-β2 was also detected as demonstrated by the results of JC-1 fluorescence measurements (Fig. 5E), which could contribute to the mitochondrial ROS generation. Co-treatment with α-klotho reduced the expression levels of ETC components, SDHB and COX1 (Fig. 5A), and restored the TGF-β2-induced superoxide production, oxygen consumption and the ΔΨm to the basal level (Fig. 5C-E). One of the main mitochondrial antioxidant enzymes, superoxide dismutase 2 (SOD2), was downregulated by TGF-β2; however, α-klotho did not recover SOD2 expression (Fig. 5A).

### Protective role of endogenous α-klotho

**Fig. 6:**
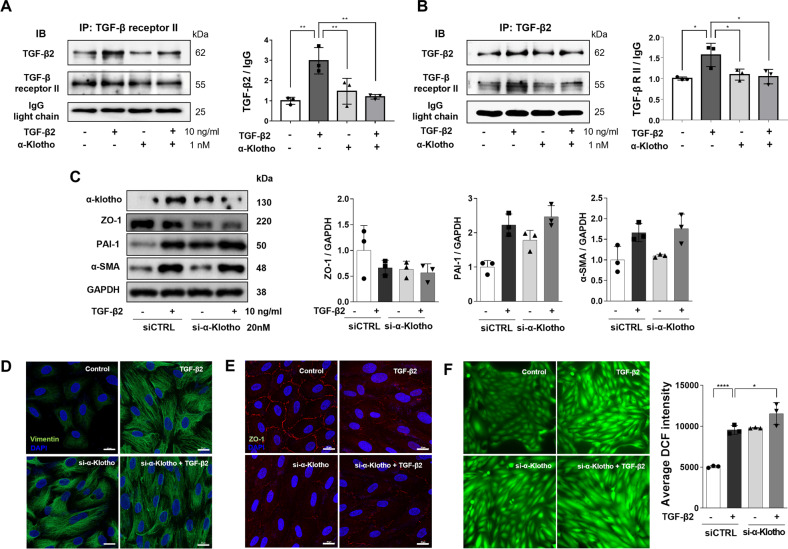
Inhibition of TGF-β2-TGF-β receptor II binding by klotho and protective role of endogenous klotho on TGF-β2-induced oxidative stress and EMT. **A, B** Immunoprecipitation experiments using TGF-β receptor II (**A**) or TGF-β2 (**B**) to detect the changes in TGF-β2 binding to TGF-β receptor II by α-klotho treatment. **C** Immunoblotting analysis for ZO-1, PAI-1 and SMA after transfection of siRNAs of scrambled sequence (siCTRL) or targeting endogenous α-klotho (*n* = 3). **D-F** Immunostaining of vimentin (**D**) and ZO-1 (**E**), and cytosolic ROS measurement using DCF fluorescence imaging analysis (*n* = 3, **F**) after siRNA transfection. Data are presented as the mean ± SE and analyzed by ANOVA. Tukey post hoc test: *p < 0.05, **p < 0.01 and ****p < 0.0001.

We investigated the molecular mechanism for the inhibitory action of α-klotho on TGF-β2-induced oxidative stress and EMT. The physical interaction between TGF-β2 and TGF-βRII was assessed using immunoprecipitation with anti-TGF-βRII antibodies followed by immunoblotting of resolved complexes using anti-TGF-β2 antibodies. The level of TGF-β2/TGF-βRII association in ARPE19 cells noticeably reduced following treatment with 1 nM α-klotho (Fig. 6A). A similar experiment using immunoprecipitation with TGF-β2 antibodies followed by immunoblotting with TGF-βRII antibodies also demonstrated α-klotho-mediated suppression of agonist-receptor binding (Fig. 6B). This evidence suggests that α-klotho inhibits TGF-β-mediated pathogenic signaling by interfering with TGF-β2 receptor binding.

To understand the role of endogenous α-klotho in RPE cells, we performed siRNA-mediated suppression of α-klotho. Silencing of α-klotho itself accelerated EMT processes such as decreased ZO-1 and increased PAI-1 and vimentin expression (Fig. 6C-E). Knockdown of α-klotho aggravates TGF-β2-induced EMT due to the loss of protective α-klotho actions. Genetic suppression of α-klotho increased oxidative stress and elevated TGF-β2-induced ROS generation, showing antioxidant consequences of endogenous α-klotho (Fig. 6F).

### α-Klotho prevents TGF-β2-induced cellular senescence in RPE cells

**Fig. 7:**
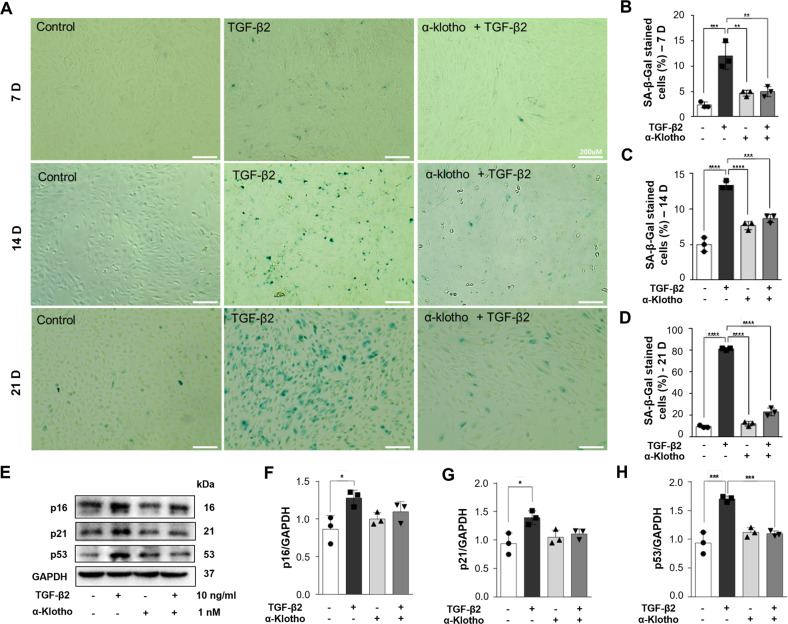
α-Klotho prevents TGF-β2-induced cellular senescence in RPE cells. **A–D** Representative β-galactosidase staining images (**A**) and quantitation of SA-β-gal-positive cells showing that α-klotho abolished the TGF-β2-induced cellular senescence in ARPE19 cells in a time-dependent manner for 7 (**B**), 14 (**C**) and 21 days (**D**). **E–H** Representative immunoblotting data (**E**) and analysis for p16 (**F**), p21 (**G**), and p53 (**H**) showing that α-klotho inhibited TGF-β2-induced cellular senescence. Scale bar 200 μm. Data are presented as the mean ± SE and analyzed by ANOVA; *n* = 3-4. Tukey post hoc test: *p < 0.05, **p < 0.01, ***p < 0.001 and ****p < 0.0001.

ARPE19 cells were incubated with TGF-β2 (10 ng/ml) for 7, 14, and 21 days to accelerate senescence to verify whether α**-**klotho inhibits TGF-β2-induced cellular senescence. Along with the morphological changes, TGF-β2 treatment resulted in a time-dependent increase in senescence-associated β-galactosidase (SA-β-Gal)-positive cells that accounted for 80% of the total cell population following TGF-β2 incubation for 21 days (Fig. 7D). However, co-incubation with α-klotho (1 nM) significantly suppressed the SA-β-Gal expression induced by TGF-β2 (Fig. 7A-D). The anti-aging effects of α-klotho were also demonstrated by the reduced expressions of senescence signaling molecules, p16, p21, and p53 (Fig. 7E-H). Taken together, soluble α-klotho protects against pathologic senescent changes of RPE mediated by suppressive actions on TGF-β2 signaling leading to oxidative stress and EMT.

## Discussion

AMD, the main cause of vision loss in the elderly, is a degenerative disorder of the central retina with multifactorial pathology. There are two types of AMD: dry (in 90% of patients) and wet (in 10% of patients), depending on the presence of choroidal neovascularization. Dry AMD is a challenging target for drug development, unlike wet AMD, because no treatment can restore the damaged RPE or photoreceptors, and the lack of suitable models for therapeutics development. The current therapeutic strategies for dry AMD highlight the importance of early treatment to delay disease progression. Here, IVT injection of TGF-β2 in mice induced characteristics of the RPE identified in patients with AMD, including morphological changes that lost their regular prismatic shape and enlarged or fused to form a non-uniform geometry with the development of stress fibers. TGF-β2-induced morphological changes were represented by increasing the cell area, perimeter, and aspect ratio and decreasing circularity. The morphological changes in RPE cells could be indicators of cellular pathophysiological status, as mouse and human cadaver eye studies reported that the size and irregularity of RPE cells increase during aging and accelerate in disease, including AMD [18–21]. The TGF-β2-induced morphological changes were also observed in in-vivo-like cell culture experiments with polarized monolayers. Exposure of ARPE19 cells, the most widely used cell line in RPE research, to TGF-β2 for one week after differentiation into polarized monolayers resulted in the loss of regular geometry and morphological changes (Supplementary Fig. S3A). Quantification represented increased cell area, perimeter, and aspect ratio with decreased circularity (Supplementary Fig. S3B). Similar morphological changes were induced in nonpolarized ARPE19 cells, and stimulation with TGF-β2 for 21 days induce more dramatic cytoskeletal organization than in vivo. Notably, aspect ratio surged 14 days after TGF-β2 stimulation, while the cell size and perimeter continued to increase during 21 days, indicating that EMT is an integral process in the progression of cellular senescence, as in the previous study [27]. α-Klotho inhibited the TGF-β2-induced morphological changes, including EMT, in vivo and in polarized and non-polarized RPE cells.

Klotho is a membrane-bound or soluble antiaging protein that protects against pathogenic stresses involved in age-related degenerative diseases [28, 29]. In this study, we observed for the first time that α-klotho prevents EMT-related degeneration and maintains epithelial integrity in the RPE. In vivo experiments showed that intravitreally injected klotho inhibited the transition to migratory mesenchymal phenotypes in EMT and increased the cell size and irregularity, which can be observed in geographic atrophy or AMD [30]. At the molecular level, klotho reversed TGF-β2-induced changes in the EMT markers and ECM remodeling proteins (MMP-2 and -9). Intriguingly, TGF-β2 can upregulate endogenously expressed α-klotho in the mouse RPE tissues and in ARPE19 cells. Moreover, exogenous α-klotho inhibited the expression of endogenous TGF-β (Fig. 3B). These results suggest that α-klotho expression in the RPE exerts a negative feedback regulation on autocrine TGF-β actions to protect against the overburden of fibrogenic and senescence-inducing stimuli.

Smad signaling is essential in the TGF-β-induced EMT process [23]. TGF-β coupling to its receptors leads to phosphorylation and activation of Smad2 and Smad3, followed by association with Smad4, a cytoplasmic co-Smad. The dimer of Smad4 with Smad2 or Smad3 translocates into the nucleus, which regulates the transcription of TGF-β target genes [31]. However, due to their low affinity for DNA, Smads require interaction with transcriptional cofactors, such as ZEB1/2, Snail1, Twist, β-catenin, and AP-1, which either promote or repress the transcription of target genes [32]. Soluble α-klotho partially inhibits the binding of TGF-β2 to its receptor, TGF-β RII, which subsequently leads to suppression of Smad2/3 phosphorylation and nuclear translocation. α-Klotho also decreased ZEB1 and β-catenin, which are key mediators of the nuclear translocation of Smads [33]. Recently, it has been reported that microRNAs are associated with the regulation of TGF-β-induced EMT in RPE cells and that miR-194 regulates ZEB1 as a functional target [34]. MiR-200c, extensively studied in the EMT of cancer cells [35], was rarely expressed in non-stimulated ARPE19 cells, while miR-200a and -429 were more abundantly expressed. MiR-200a and miR-429 reduced by TGF-β2 were either fully recovered or increased compared to the baseline levels by α-klotho, leading to normalization of the transcriptional level of ZEB1. The knockdown of ZEB1 prevented the morphological alterations induced by miR-200a inhibitors, implying that the miR-200a-ZEB1 axis plays an essential role in regulating cellular morphological features. Since these regulations were not noticeably changed by α-klotho co-treatment, we suggest that α-klotho is an upstream regulator of the miR-200a-ZEB1 axis, leading to the prevention of senescence-like morphological changes, including pathological EMT of the RPE.

Our previous reports demonstrated that ERK1/2-mTORC1 activation by TGF-β1 induces NOX4 upregulation and cytosolic ROS generation in ARPE19 cells [24]. TGF-β2 also activates ERK1/2-mTORC1 signaling and upregulates NOX4, which accounts for cytosolic oxidative stress (Fig. 3A). It has been demonstrated that TGF-β-induced actin cytoskeleton rearrangement is accompanied by compensatory upregulation of mitochondrial oxidative phosphorylation [36]. TGF-β-stimulated oxidative phosphorylation increases the ΔΨ_m_, which is known to elevate the generation of mitochondrial superoxide [26]. Indeed, we also detected mitochondrial ROS generation induced by TGF- β2 associated with increased ETC expression, basal mitochondrial respiration and proton leak, and hyperpolarization of the ΔΨ_m_. α-Klotho prevented TGF-β2-induced mitochondrial activation and superoxide generation. It is known that α-klotho suppresses oxidative stress by upregulating SOD2 via FOXO activation [37, 38]. In ARPE19 cells, TGF-β2 markedly inhibited SOD2 expression, leading to further oxidative damage (Fig. 4A). However, α-klotho neither increased SOD2 expression nor prevented TGF-β2-induced SOD2 downregulation, which is different from previous results in other tissues.

In conclusion, IVT injection of α-klotho attenuates TGF-β2-induced degenerative changes of the RPE by inhibiting EMT and oxidative stress. As a potential mechanism, we provided evidence that α-klotho inhibits TGF-β receptor binding, decreases cytosolic and mitochondrial oxidative stress by attenuating NOX4 and mitochondrial respiratory activities, and relieves the miR-200a-ZEB1 axis suppression related to EMT induction. Our results imply that soluble α-klotho is a potential therapeutic agent for maintaining the epithelial integrity of the RPE and delaying dry AMD progression. Further studies will be needed to investigate the complex mechanism underlying the effects of α-klotho on dry AMD retardation and its long-term effects in vivo. However, α-klotho, as an EMT-targeting therapy, may be a promising therapeutic agent against dry AMD that can restore the epithelial phenotype and ensure long-term drug efficacy.

## Materials and Methods

### Animals

This study conformed to the Guide for the Care and Use of Laboratory Animals published by the United States National Institutes of Health (NIH Publication, 8th edition, 2011). All animal care and experimental procedures were approved by the Institutional Animal Care and Use Ethics Committee of Seoul National University (No. SNU-190814-2), and all efforts were made to minimize animal suffering and reduce the number of animals used. Six-week-old male C57BL/6 J mice (18-22 g, Orient Bio Inc, Sungnam, Korea) were housed in an animal care facility at a controlled environment temperature (21 ± 2 °C) and humidity (45 ± 15%) and provided with free access to standard rodent chow and water. All animals were allowed to acclimate for at least seven days before the experiments and subjected to a 12-hr light-dark schedule. The mice were randomly allocated into experimental groups for the immunofluorescence analysis, immunoblotting analysis, and toxicity assessment groups, with each group consisting of five mice based on body weight. Animals were anesthetized by intraperitoneal injection of a mixture of Zoletil 50® (50 mg/kg) and xylazine (5 mg/kg). Before the injection, the animals received tropicamide (Tropherin®) eye drops for dilatation. A step incision in the pars plana region of the sclera was made using a 26 G needle, and intravitreal (IVT) injections were carried out using a Hamilton syringe (Hamilton, Bonaduz, Switzerland) under a surgical microscope (Leica Microsystems. Ltd., Wetzlar, Germany). The animals received an IVT injection of 1 µg of recombinant mouse TGF-β2 (# 7346-B2-005, R&D Systems) with or without 10 fmoles of α-klotho. Recombinant human α-klotho (# 5334-KL-025, R&D Systems, Minneapolis, MN, USA) was dissolved in PBS solution containing 50% glycerol, 0.1 mM EDTA, and 0.5 mM dithiothreitol (pH 6.0). The stock solution (1 μM) was aliquoted and stored at −80 °C.

### RPE-choroid-scleral complex processing and immunohistochemistry

Three days after the injection, the animals were sacrificed, and their eyes were fixed by immersion in 4% paraformaldehyde for 15 min at room temperature. The RPE-choroid-scleral complexes (RCSs) were carefully separated from the eyeball using a dissecting microscope (Nikon SM2745T, Nikon Corporation, Japan) and incubated with Perm/Block solution (0.2% Triton-X 100 and 0.3% BSA in PBS). Subsequently, they were incubated overnight at 4 °C with Alexa Fluor 594-conjugated anti-zonula occludens (ZO)-1 (# 339194, Invitrogen, Carlsbad, CA, USA) and Alexa Fluor 488-conjugated anti-phalloidin (#: A12379, Invitrogen, Carlsbad, CA, USA) antibodies, and counterstained with 10 mg/ml DAPI (Sigma Aldrich). After washing with PBS, the RPE-choroid complexes were mounted using Fluoromount^TM^ Aqueous Mounting Medium (DAKO, Glostrup, Denmark) and observed under a confocal microscope (Leica TCS STED, Leica Microsystem Ltd., Wetzlar, Germany). Six z-stack images were taken from the central and equatorial regions of the RCSs in each eye. The size, perimeter, aspect ratio (major axis/minor axis), and circularity [4π(area/perimeter)^2^] of RPE cells were measured manually using the ImageJ software based on the ZO-1 stained RPE cell margin [18, 20]. ARPE19 cells were washed with PBS after removal of the medium and fixed with 4% paraformaldehyde for 15 min at room temperature. Cells were incubated with an Alexa Fluor 488 conjugated anti-phalloidin antibody (#: A12379, Invitrogen, Carlsbad, CA, USA) overnight at 4 °C. Nuclei were counterstained with DAPI for 5 min at room temperature. After washing with PBS, cells were mounted using Fluoromount^TM^ Aqueous Mounting Medium (DAKO, Glostrup, Denmark) and five microscopic images were taken in each group using a fluorescence microscope (Nikon Eclipse 80i, Nikon Corporation, Japan). We analyzed four cellular morphometric parameters, including cell area, perimeter, aspect ratio, and circularity, from images obtained via confocal microscopy, according to the Image J user guidelines. We excluded the cells at the image edges and those that could not be fully identified. The spatial scale of the images was defined so that the measurement result could be displayed in calibrated units in μm. We calculated the area (μm^2^) and length (μm) of the selected region by selecting the outside boundary of the f-actin or ZO-1-stained each cell. Aspect ratio and circularity were calculated using the shape descriptor of Image J in the selected cell area. Aspect ratio (major axis/minor axis) is calculated as the ratio between the major and minor axes of an ellipse, and this parameter is higher in elongated cells. Circularity ([4π(area/perimeter)^2^]) is a shape descriptor that mathematically indicates the degree of similarity to a perfect circle, with a value close to 1.0 designating a perfect circle and a value close to 0.0 indicating a less circular shape.

### Cell culture

Adult retinal pigment epithelial cell line-19 (ARPE19) cells (American Type Culture Collection, Manassas, VA, USA) were maintained in Dulbecco’s modified Eagle’s medium/Ham’s F12 (SH30023.01, Thermo Fisher Scientific, Inc., Waltham, MA, USA) supplemented with 10% fetal calf serum (10099141, Gibco, Grand Island, NY, USA). The cells were grown at 37 °C in 5% CO_2_ and 95% air. TGF-β2-stimulation was performed using recombinant human TGF-β2 (# 302-B2, R&D systems) as previously described [12]. Fresh growth medium was added to the cells every three to four days until confluence. Both unstimulated and TGF-β2-treated cells were split once a week at a ratio of 1:5 with Dulbecco’s modified Eagle’s medium/Ham’s F12 with 5% fetal calf serum to retain cell viability for 21 days. Three independent time courses were conducted.

### Immunocytochemistry

ARPE19 cells grown on 18 mm coverslips were washed twice with PBS and fixed with 4% paraformaldehyde for 15 min at 37 °C. Next, the cells were permeabilized with 0.25% Triton X-100 for 5 min and blocked with 1% BSA in PBS for 30 min. Incubation with Alexa Fluor™ 594 Phalloidin (1:100, #A12381, Invitrogen, Carlsbad, CA, USA), ZO-1 (1:50, #61-7300, Invitrogen), and vimentin (1:00, #5741, Cell Signaling Technology, Beverly, MA, USA) antibodies was performed overnight at 4 °C, followed by incubation with a secondary antibody for 1 h at room temperature in a dark room. Cells were washed and counterstained with 1 μg/ml 4’,6’-diamidino-2-phenylindole (DAPI) for 5 min. Fluorescence images were obtained using a laser scanning confocal microscope (LSM800, Zeiss, Oberkochen, Germany).

### Western blotting

The pooled protein was extracted from the RPE-choroid scleral complexes in five mice from each group using RIPA buffer (1% Triton X-100). Protein extracts had also prepared using RIPA buffer (1% Triton X-100) in ARPE19 cells from three independent experiments. The samples were then centrifuged and the supernatants were collected. The protein content was measured using the BCA protein assay kit (Pierce, Rockford, IL, USA). Proteins were separated using SDS-PAGE and transferred onto polyvinylidene fluoride (PVDF) membranes (Merck Millipore Ltd., Tullagreen, Ireland). The PVDF membrane was blocked using non-fat milk and incubated with TBST. The antibodies used for western blotting are summarized in Supplementary Table S1. Immunoreactive bands were visualized using ImageQuant^TM^ LAS4000 (GE Healthcare, USA) according to the manufacturer’s instructions. Densitometric analysis was performed using the ImageJ software. The results were normalized to those of β-actin. Both nuclear and cytoplasmic extracts from ARPE19 cells were prepared using an NE-PER Nuclear and Cytoplasmic Extraction Reagent kit (Pierce, Rockford, IL, USA) according to the manufacturer’s instructions for the analysis of Smad4 and ZEB1.

### Senescence-associated β-galactosidase (SA- β-Gal) staining

Senescence-associated β-Galactosidase (SA-β-Gal) staining was performed as previously described [39]. Briefly, ARPE19 cells were washed with PBS, fixed with 3.7% paraformaldehyde for 2 min, and then incubated for 24 h at 37 °C with the SA-β-Gal staining solution of the Senescence β-Galactosidase Staining Kit (Cell signaling Technology, Beverly, MA, USA) according to the manufacturer’s instructions. SA-β-Gal densities (% area) were determined using the ImageJ software based on five microscopic images in each group.

### Cytosolic and mitochondrial reactive oxygen species (ROS) measurement

Cytosolic ROS generation was detected using chloromethyl-H_2_-dichlorofluorescein diacetate (CM-H_2_DCF-DA), which is rapidly oxidized to highly fluorescent 2,7-dichlorofluorescein (DCF) by intracellular ROS. ARPE19 cells cultured on 12 mm coverslips were treated with a 5 μM working concentration of CM-H_2_DCF-DA for 20 min at 37 °C and rinsed with Krebs-Ringer bicarbonate (KRB) solution (135 mM NaCl, 3.6 mM KCl, 2 mM NaHCO_3_, 0.5 mM NaH_2_PO4, 0.5 mM MgSO_4_, 1.5 mM CaCl_2_, and 10 mM HEPES; pH 7.4). Mitochondrial superoxide generation was detected using mitoSox (Molecular Probes), a red fluorescent dye localized to the mitochondria. Once it enters the mitochondria, mitoSox is specifically oxidized by superoxide and exhibits red fluorescence. MitoSox (5 μM) was used to load ARPE19 cells for 20 min and then, the cells were washed twice with KRB solution. Fluorescence images (excitation/emission: 490/535 nm for DCF and 514/560 nm for mitoSox) were captured using a microscope (IX81, Olympus, Tokyo, Japan), and the intensity was analyzed using the Metamorph 6.1 software (Molecular Devices, Sunnyvale, CA).

### Mitochondrial oxygen consumption rate (OCR) measurement

ARPE19 cells were plated on Seahorse 96 well plates (Agilent Technologies, Cedar Creek, TX, USA) and cultured for 48 h with TGF-β2 or Rg3. After culturing, the cells were changed to pre-warmed XF DMEM medium (for OCR measurement, containing 1 M glucose, 100 mM pyruvate and 200 mM L-glutamate and adjusted at pH 7.4; Agilent Technologies) 1 h prior to analyses and kept cells at 37 °C without CO_2_. OCR was measured in human bone marrow stem cells (hBMSCs) using the Seahorse XFe96 Analyzer (Agilent Technologies) to determine the oxygen consumption in response to the addition of various chemicals [40]. The cycles (3 times for 3 min) were run for every measurement and the Mitostress kit (Agilent Technologies), containing the following compounds: 2 μM oligomycin (ATP synthase inhibitor), 2 μM carbonyl cyanide-4-(trifluoromethoxy) phenylhydrazone (FCCP) (mitochondrial uncoupler), 0.5 μM rotenone (respiratory chain complex I inhibitor), and antimycin A (complex III inhibitor), was used. The OCR was normalized based on the protein concentration determined using the BCA protein assay.

### Mitochondrial membrane potential measurement

The mitochondrial membrane potential (ΔΨ_m_) of ARPE19 cells was measured using the lipophilic cationic dye 5,5′,6,6′-tetrachloro-1,19,3,39-tetraethylbenzimidazolyl-carbocyanine iodide (JC-1) (Molecular Probes, Invitrogen). JC-1 monomers enter the mitochondria based on the ΔΨ_m_ and form J-aggregates inside the mitochondria, transmitting red fluorescence (excitation/emission wavelength: 540/590 nm), whereas the rest of the monomers transmit green fluorescence (excitation/emission wavelength: 490/540 nm). The ratio of red/green fluorescence was used as a ΔΨ_m_ indicator. ARPE19 cells were grown in 96-well flat, clear bottom, black-walled polystyrene TC-treated microplates (Corning Incorporated, Corning, NY, USA). Cells were washed twice with the KRB solution after loading with JC-1 (300 nM) for 40 min, and the fluorescence was recorded using a fluorescence microplate reader (FlexStation II, Molecular Devices), as previously described [41].

### Immunoprecipitation

Protein samples from cell lysates were precleared using 50 µL of packed protein A-crosslinked 4% beaded agarose (Cell Signaling Technology, Beverly, MA, USA) at 4 °C for 1 h. The beads were removed using centrifugation and the supernatant was collected. The supernatant was incubated with an antibody specific for TGF-β receptor II (TGF-β RII)(1:1000, ab225902, Abcam, Cambridge, UK) at 4 °C with constant mixing for 12 h. The immune complex was captured following the addition of packed agarose protein A beads (50 µL/ 500 µL supernatant) for 2 h at 4 °C. Bead separation was achieved using centrifugation (13,000× *g* for 25 min) and the supernatant was removed. Subsequently, the samples were subjected to immunoblot analysis with specific primary antibodies, which are summarized in Supplementary Table S1. Secondary anti-rabbit (1:2000, #3678, Cell Signaling Technology, Beverly, MA, USA) and anti-rat IgGs (1:2000, sc2006, Santa Cruz, Dallas, Texas, USA respectively) were used to visualize the immunoreactive bands. The specificity of immunoprecipitation was confirmed using negative control reactions performed with either no primary antibody or rabbit IgG control.

### Quantification of miRNA and mRNA

Total RNA was extracted using the Trizol reagent (Invitrogen, Paisley, UK) according to the manufacturer’s instructions. Primers for E-cadherin (Hs01023895_m1), ZEB1 (Hs01566408_m1), and GAPDH (Hs99999905_m1) were obtained from Applied Biosystems, and the results were normalized to GAPDH expression and further normalized to the non-stimulated control. For mRNA analysis, cDNA synthesis was performed using the TaqMan MicroRNA Reverse Transcription Kit (Applied Biosystems, Foster City, CA, USA) and RT-primers for miR-200a, miR-200b, miR-200c, miR-141, miR-429, and cel-miR-39 purchased from Applied Biosystems (Foster City, CA, USA). The results were normalized to those of exogenous cel-miR-39 and further normalized to those of cells non-stimulated for seven days.

### Transfection of miRNA inhibitors and siRNA

ARPE19 cells were seeded at a density of 6 × 10^4^ cells per well in 24-well plates and transfected with 100 nM of miR-200a inhibitors or a negative control inhibitor (Dharmacon, Lafayette, Colorado, USA) using the Lipofectamine^®^ RNAiMAX reagent (Invitrogen, Paisley, UK). After three days of transfection, the cells were split and re-transfected with additional miR-200a inhibitors. ZEB1 was knocked down using 10 nM siRNA or control siRNA (Invitrogen, Paisley, UK). This process was repeated every three to four days for up to 21 days [12]. Immunofluorescence staining of F-actin was performed at 7, 14, and 21 days post-transfection. For genetic suppression experiments, siRNAs for α-klotho and scrambled sequence (control) were purchased from Santa Cruz Biotechnology (sc-43883, Santa Cruz, CA, USA) and transfected with DharmaFECT (Dharmacon).

### Statistical Analysis

The results are presented as the mean ± SEM. We used one-way ANOVA analysis followed by Tukey post hoc test (for three or more data samples) to explore the differences in the morphological changes and oxidative stress of ARPE19 cells induced by TGF-β2 and its attenuation by α-klotho. No statistical methods were used to predetermine the sample size. Mice were randomly allocated to experimental groups. No blinding method was used for IVT injection. There were no animal exclusion criteria. The variance was similar between the groups that were being statistically compared. One-way ANOVA and Tukey analysis were performed with GraphPad Prism (San Diego, CA) version 7.0 software. Data were considered statistically significant at *p* < 0.05.

## Supplementary information

Checklist

Supplementary Figures & Table

Original Data File

## Data Availability

The data that support the findings of this study are available from the corresponding author upon reasonable request.
