# Supplementary material for: Klotho prevents transforming growth factor-β2-induced senescent-like morphological changes in the retinal pigment epithelium
Source: Cell Death Dis. 2023 May 20;14(5):334. doi: 10.1038/s41419-023-05851-8 (PMC10199917; doi:10.1038/s41419-023-05851-8)
Supplement: Supplementary file 2 — Supplementary Figures & Table [file 41419_2023_5851_MOESM2_ESM.docx]

## **Klotho prevents transforming growth factor-β2-induced senescent-like morphological changes in the retinal pigment epithelium**

**Authors**

Ha Young Jang^1*^, Soo-Jin Kim^2,3*^, Kyu-Sang Park^2,3‡^, Jeong Hun Kim^1,4,5‡^

**Affiliations**

^1^Fight against Angiogenesis-Related Blindness (FARB) Laboratory, Clinical Research Institute, Seoul National University Hospital, Seoul, Republic of Korea;

^2^Department of Physiology, Yonsei University Wonju College of Medicine, Wonju, Republic of Korea;

^3^Mitohormesis Research Center, Yonsei University Wonju College of Medicine, Wonju, Republic of Korea;

^4^Department of Ophthalmology, College of Medicine, Seoul National University, Seoul, Republic of Korea;

^5^Institute of Reproductive Medicine and Population, Seoul National University College of Medicine, Seoul, Republic of Korea

Supplementary Figures S1 to S3

Supplementary Table S1


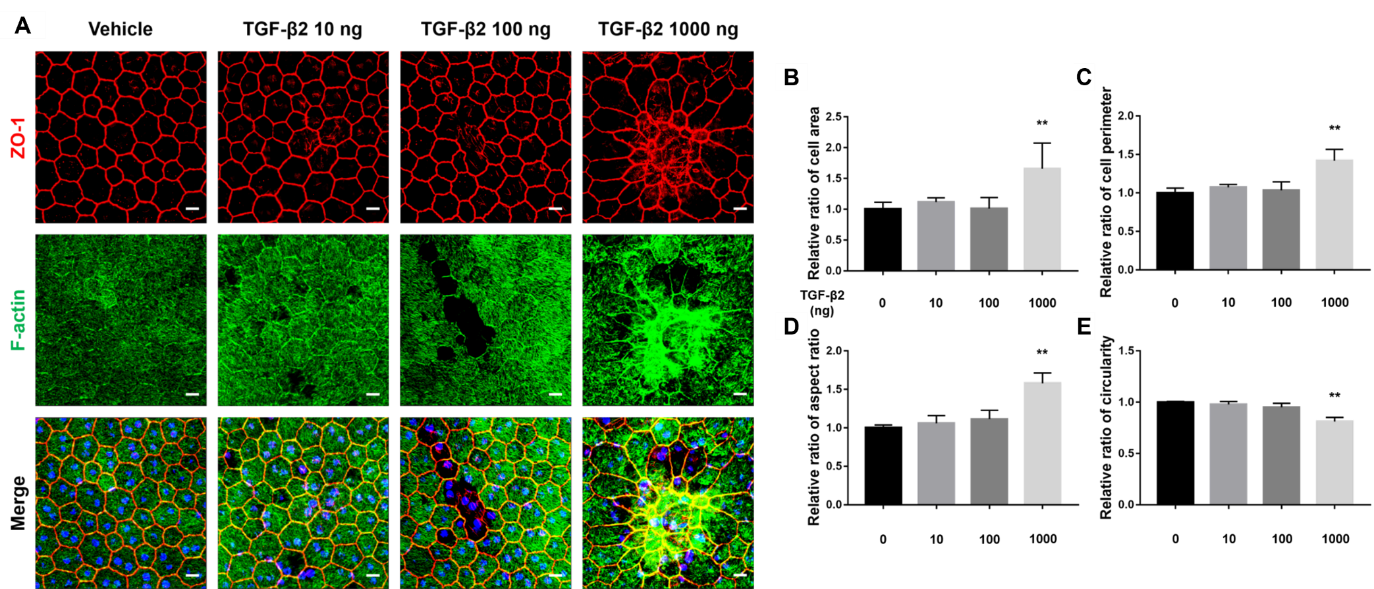


**Supplemental Fig. S1. Induction of morphological changes in the RPE by IVT injection of TGF-β2 at a dose of 1000 ng in mice.** Mice received IVT injection (2 μl) with vehicle (PBS), 10 ng, 100 ng, or 1000 ng of mouse recombinant TGF-β2. Three days after the injection, all mice were sacrificed. **A** Representative immunofluorescence images of the RPE stained for ZO-1 and F-actin (n=5). **B** Morphological analysis of the relative ratio of cell area. **C** Morphological analysis of the relative ratio of cell perimeter. **D** Morphological analysis of the relative ratio of aspect ratio. **E** Morphological analysis of the relative ratio of circularity. Scale bar 10 μm. Data from three experiments (mean ± SE) with n=5 mice per group and were analyzed by ANOVA. Tukey post hoc test: **p*<0.05. ***p*<0.01.


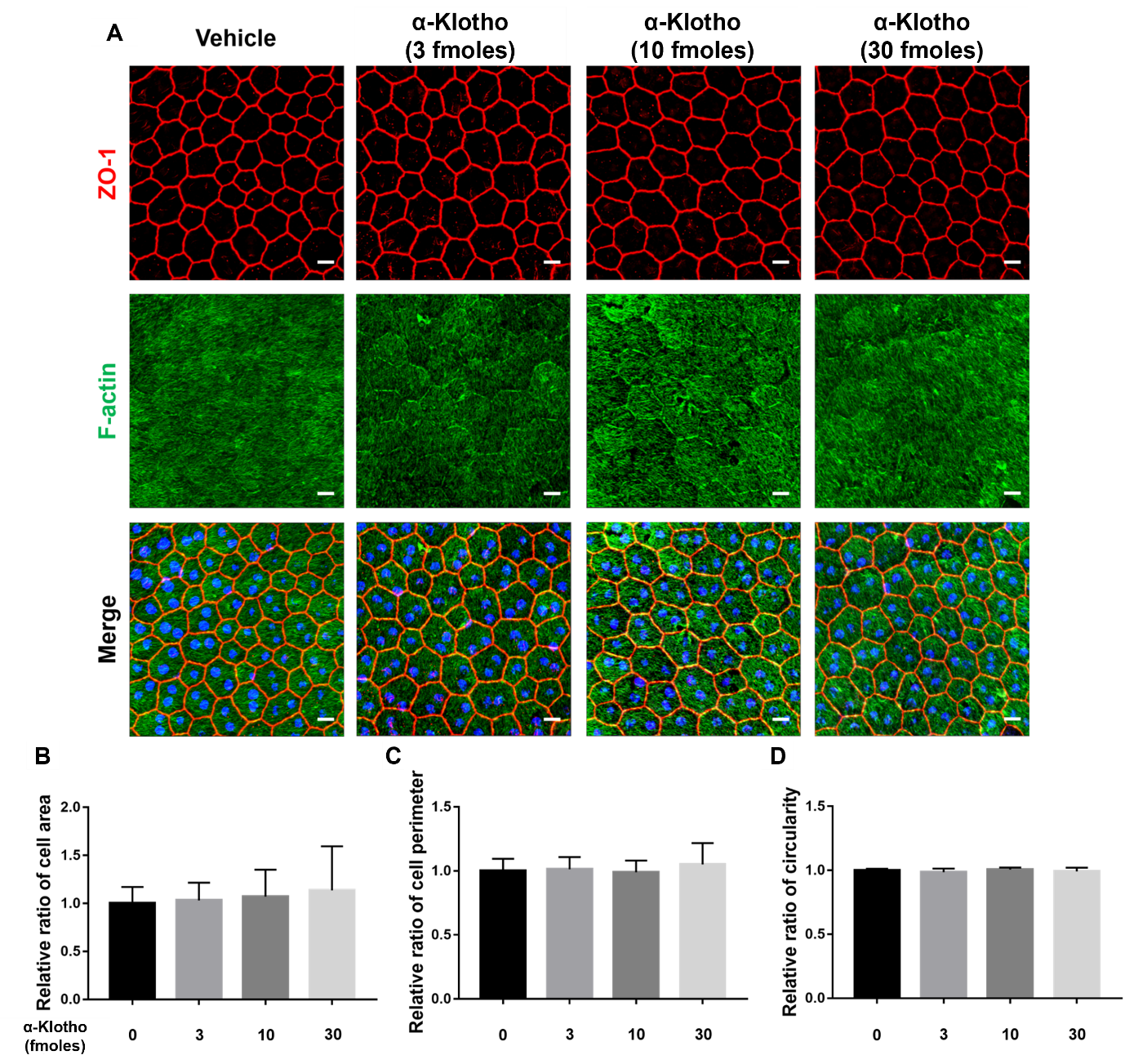


**Supplemental Figure S2. IVT injection of α-klotho did not induce significant morphological changes in the mice RPE.** Mice received IVT injection (2 μl) with vehicle (PBS), 3 fmoles, 10 fmoles, or 30 fmoles of α-klotho. Three days after the injection, all mice were sacrificed. **A** Representative immunofluorescence images of the RPE stained for ZO-1 and F-actin (n=5). **B** Morphological analysis of the relative ratio of cell area. **C** Morphological analysis of the relative ratio of cell perimeter. **D** Morphological analysis of the relative ratio of circularity. Scale bar 10 μm. Data from three experiments (mean ± SE) with n=5 mice per group and were analyzed by ANOVA. Tukey post hoc test: **p*<0.05. ***p*<0.01.


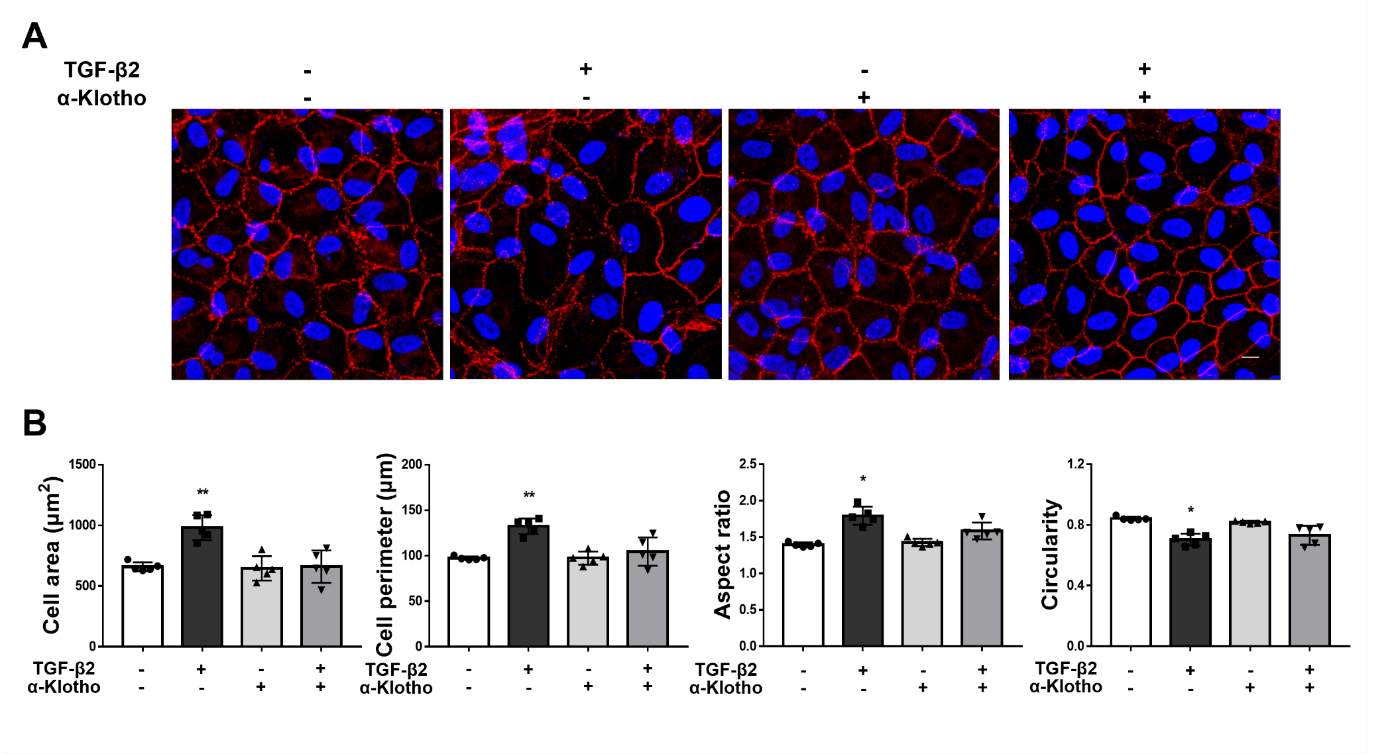


**Supplemental Figure S3. Morphological changes induced by TGF-β2 and the attenuation by α-klotho in polarized monolayer ARPE19 cells.** After differentiation into a polarized monolayer, ARPE19 cells were treated with TGF-β2 (10 ng/ml), α-klotho (1 nM), or both for one week. The cell area, perimeter, and aspect ratio were significantly increased, and circularity was reduced by TGF-β2 treatment, as observed in non-polarized ARPE19 cells and *in vivo* mice. The observed morphological changes were significantly attenuated by α-klotho co-treatment. **A** Representative immunofluorescence images of ARPE19 cells stained for ZO-1. **B** Morphological analysis of cell size (μm^2^). **C** Morphological analysis of cell perimeter (μm). **D** Morphological analysis of aspect ratio. **E** Morphological analysis of circularity. Scale bar 10 μm. Data from five experiments (mean ± SE) are shown in bars and were analyzed by ANOVA. Tukey post hoc test: **p*<0.05. ***p*<0.01.

| **Antibody** | **Company** | **Catalog No.** | **RRID** | **Dilution** |
| --- | --- | --- | --- | --- |
| TGF-β | R&D | MAB1835 | AB_357931 | 1:250 (WB) |
| TGF-β2 | Abcam | ab113670 | AB_11127268 | 1:1000 (WB) |
| TGF-βR II | Abcam | Ab225902 | - | 1:1000 (WB) |
| ZEB1 | Abcam | ab81972 | AB_1658297 | 1:1000 (WB) |
| Smad4 | Cell Signaling | 46535 | AB_2736998 | 1:1000 (WB) |
| β-actin | Cell Signaling | 4967 | AB_330288 | 1:1000 (WB) |
| MMP-9 | Santa Cruz | sc-393859 | - | 1:500 (WB) |
| p-ERK1/2 | Cell Signaling | 4370 | AB_2315112 | 1:1000 (WB) |
| t-ERK1/2 | Cell Signaling | 9102 | AB_330744 | 1:1000 (WB) |
| α-klotho | Trans Genic | KO603 | AB_2722757 | 1:500 (WB) |
| SOD2 | Cell Signaling | 13194 | AB_2750869 | 1:1000 (WB) |
| p-70S6K | Cell Signaling | 9205 | AB_330944 | 1:1000 (WB) |
| t-70S6K | Cell Signaling | 9202 | AB_331676 | 1:1000 (WB) |
| p-Smad3 (Ser423/425) | Cell Signaling | 9520 | AB_2193207 | 1:1000 (WB) |
| t-Smad3 | Cell Signaling | 9523 | AB_2193182 | 1:1000 (WB) |
| p-Smad2 | Cell Signaling | 3108 | AB_490941 | 1:1000 (WB) |
| t-Smad2 | Cell Signaling | 5339 | AB_10626777 | 1:1000 (WB) |
| α-SMA | Abcam | ab5694 | AB_2223021 | 1:1000 (WB) |
| Anti-Active-β-Catenin (Anti-ABC) | MERCK | 05-665 | AB_309887 | 1:1000 (WB) |
| SDHB | Santa Cruz | sc-25851 | AB_2183458 | 1:1000 (WB) |
| COX1 | Invitrogen | 459600 | - | 1:1000 (WB) |
| PAI-1 | Thermo scientific | PA5-27216 | AB_2544692 | 1:1000 (WB) |
| NOX4 | Novus | NB110-58849 | AB_877739 | 1:1000 (WB) |
| ZO-1 | Cell Signaling | 8193 | AB_10898025 | 1:1000 (WB) |
| E-cadherin | Cell Signaling | 3195 | AB_2291471 | 1:1000 (WB) |
| p15/p16 | Santa Cruz | sc-377412 | - | 1:1000 (WB) |
| p21 | Santa Cruz | sc-6246 | AB_628073 | 1:1000 (WB) |
| p53 | Santa Cruz | sc-126 | AB_628082 | 1:1000 (WB) |
| GAPDH | Santa Cruz | sc-25778 | AB_10167668 | 1:5000 (WB) |
| Anti-rabbit 2° Ab, HRP conjugate | Thermo scientific | 31460 | AB_228341 | 1:5000 (WB) |
| Anti-mouse 2° Ab, HRP conjugate | Thermo scientific | 31450 | AB_2548924 | 1:5000 (WB) |
| Anti-rabbit 2° Ab, HRP conjugate | Cell Signaling | 3678 | AB_1549606 | 1:2000 (WB) |
| Anti-mouse 2° Ab, HRP conjugate | Santa Cruz | sc-2006 | AB_1125219 | 1:2000 (WB) |
| Anti-goat 2° Ab, HRP conjugate | Santa Cruz | Sc-2354 | AB_628490 | 1:2000 (WB) |
| Alexa Fluor™ 488 ZO-1 | Invitrogen | 61-7300 | AB_138452 | 1:50 (IF) |
| Alexa Fluor™ 594 Phalloidin | Invitrogen | A12381 | AB_2315633 | 1:100 (IF) |
| Alexa Fluor™ 594 ZO-1 | Invitrogen | 339194 | AB_2532188 | 1:100 (IF) |
| Alexa Fluor™ 488 Phalloidin | Invitrogen | A12379 | SCR_004098 | 1:100 (IF) |
| Vimentin | Cell Signaling | 5741 | AB_10695459 | 1:100 (IF) |
| Goat anti-Rabbit IgG(H+L), Alexa Fluor 488 | Invitrogen | A-11008 | AB_143165 | 1:400 (IF) |
| Goat anti-Rabbit IgG(H+L), Alexa Fluor 594 | Invitrogen | A-11012 | AB_2534079 | 1:400 (IF) |

**Supplementary Table S1. List of antibodies**
