# Supplementary material for: Klotho prevents transforming growth factor-β2-induced senescent-like morphological changes in the retinal pigment epithelium
Source: Cell Death Dis. 2023 May 20;14(5):334. doi: 10.1038/s41419-023-05851-8 (PMC10199917; doi:10.1038/s41419-023-05851-8)
Supplement: Supplementary file 3 — Original Data File [file 41419_2023_5851_MOESM3_ESM.pptx]

## Slide 1
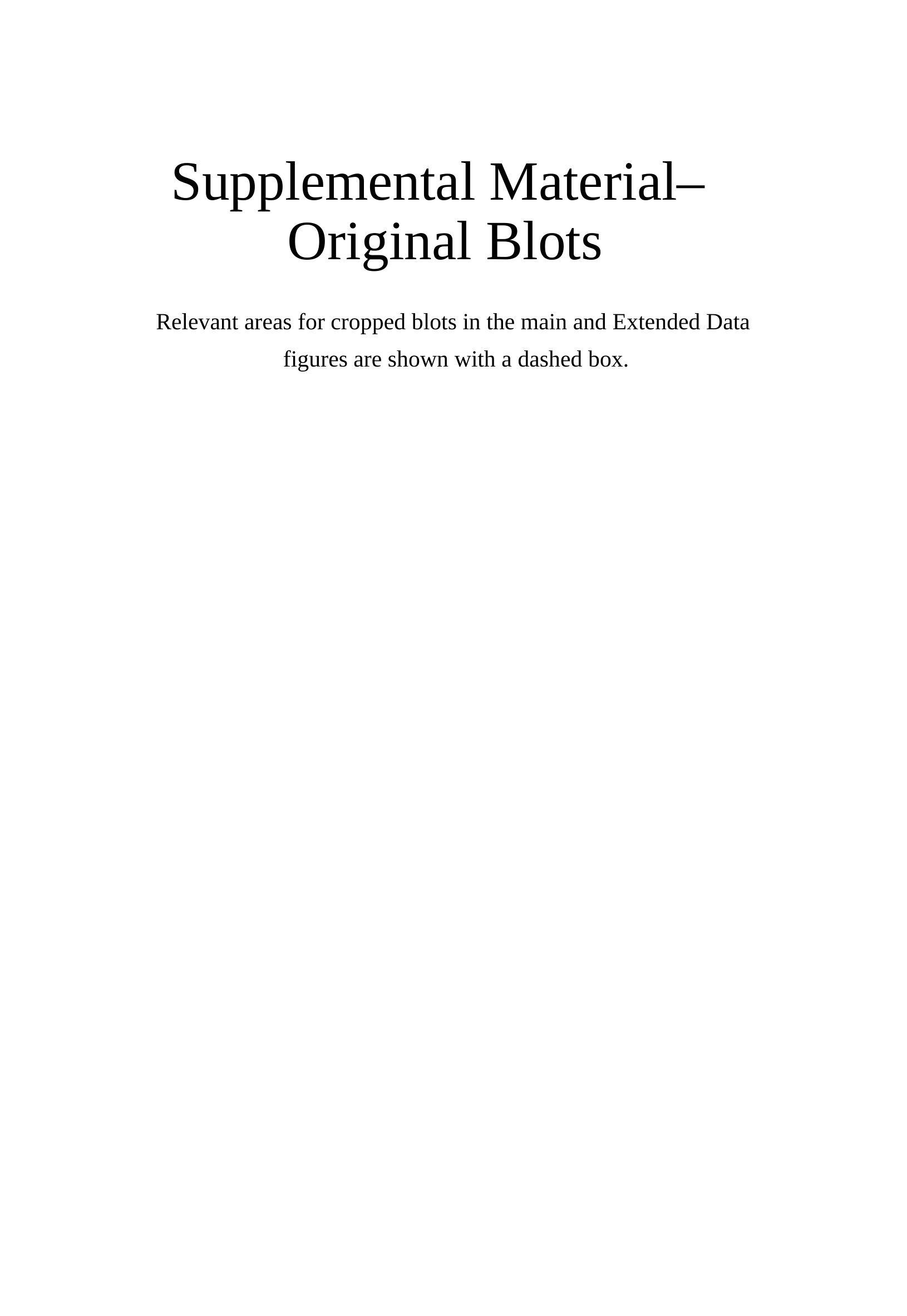

Supplemental Material–
Original Blots
Relevant areas for cropped blots in the main and Extended Data
figures are shown with a dashed box.

## Slide 2
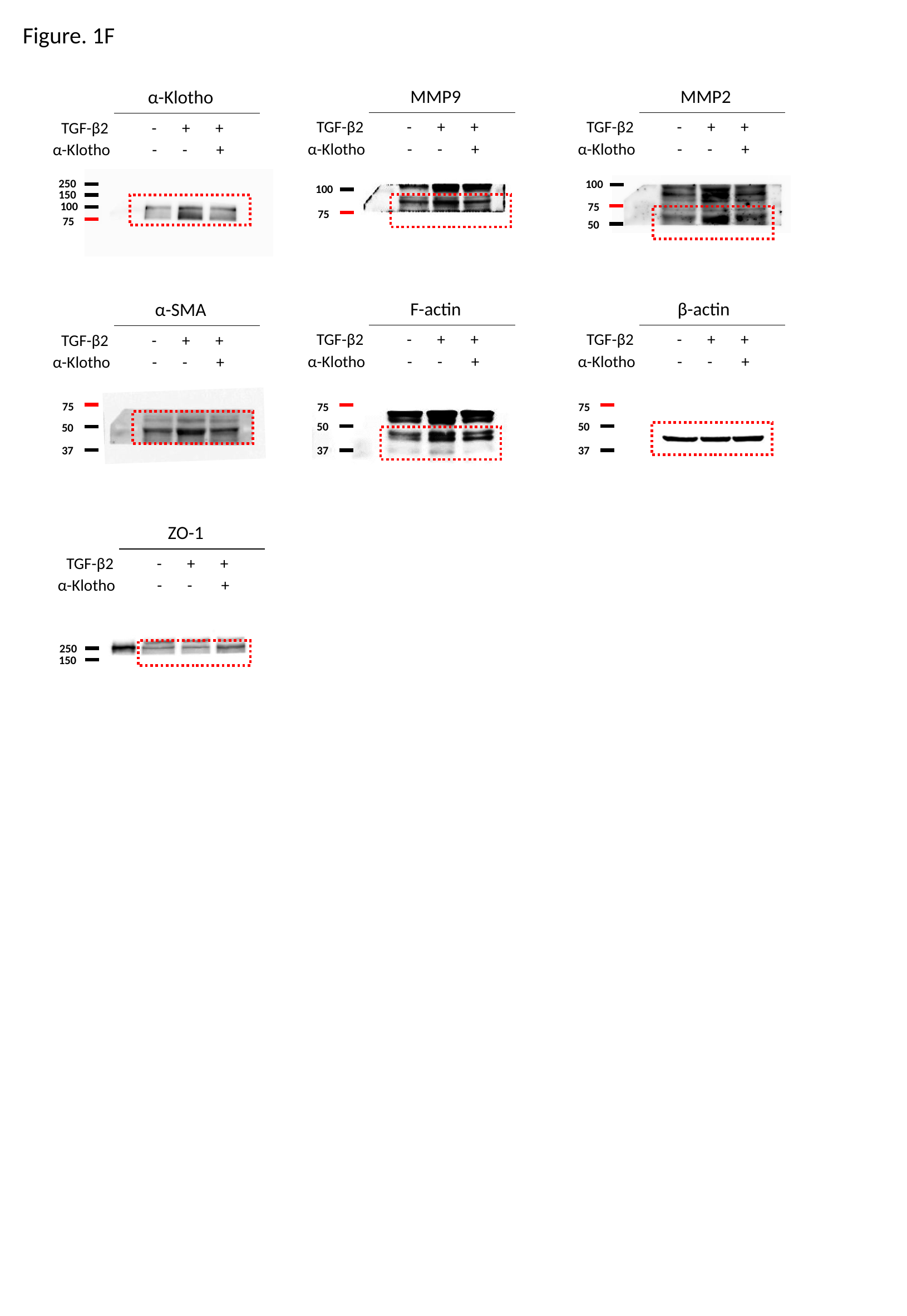

Figure. 1F
MMP9
MMP2
α-Klotho
TGF-β2 - + +
TGF-β2 - + +
TGF-β2 - + +
α-Klotho - - +
α-Klotho - - +
α-Klotho - - +
250
100
100
150
100
75
75
75
50
F-actin
β-actin
α-SMA
TGF-β2 - + +
TGF-β2 - + +
TGF-β2 - + +
α-Klotho - - +
α-Klotho - - +
α-Klotho - - +
75
75
75
50
50
50
37
37
37
ZO-1
TGF-β2 - + +
α-Klotho - - +
250
150

## Slide 3
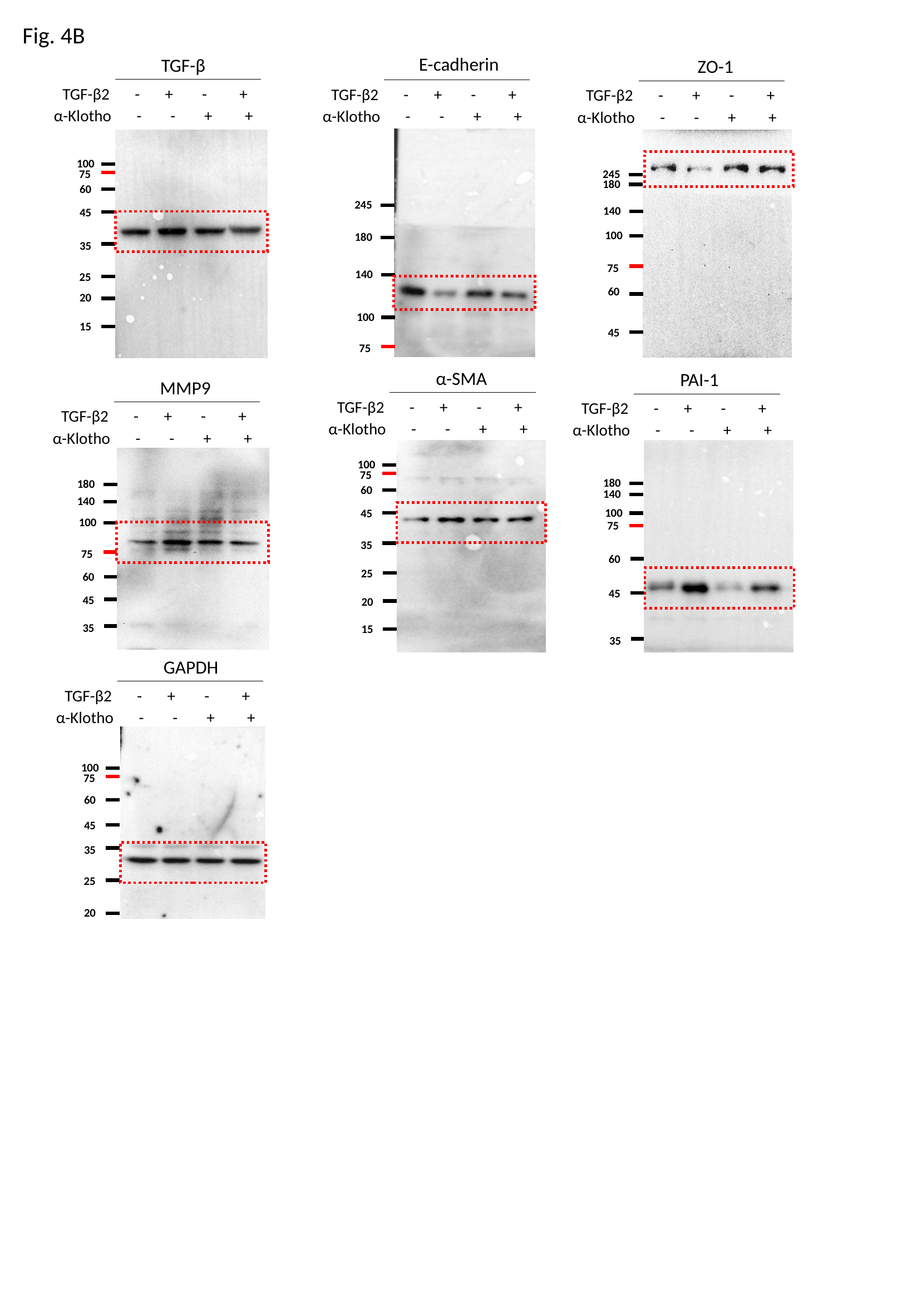

Fig. 4B
E-cadherin
TGF-β
ZO-1
TGF-β2 - + - +
TGF-β2 - + - +
TGF-β2 - + - +
α-Klotho - - + +
α-Klotho - - + +
α-Klotho - - + +
100
75
245
180
60
245
140
45
100
180
35
75
140
25
60
20
100
15
45
75
α-SMA
PAI-1
MMP9
TGF-β2 - + - +
TGF-β2 - + - +
TGF-β2 - + - +
α-Klotho - - + +
α-Klotho - - + +
α-Klotho - - + +
100
75
180
180
60
140
140
100
45
100
75
35
75
60
25
60
45
45
20
35
15
35
GAPDH
TGF-β2 - + - +
α-Klotho - - + +
100
75
60
45
35
25
20

## Slide 4
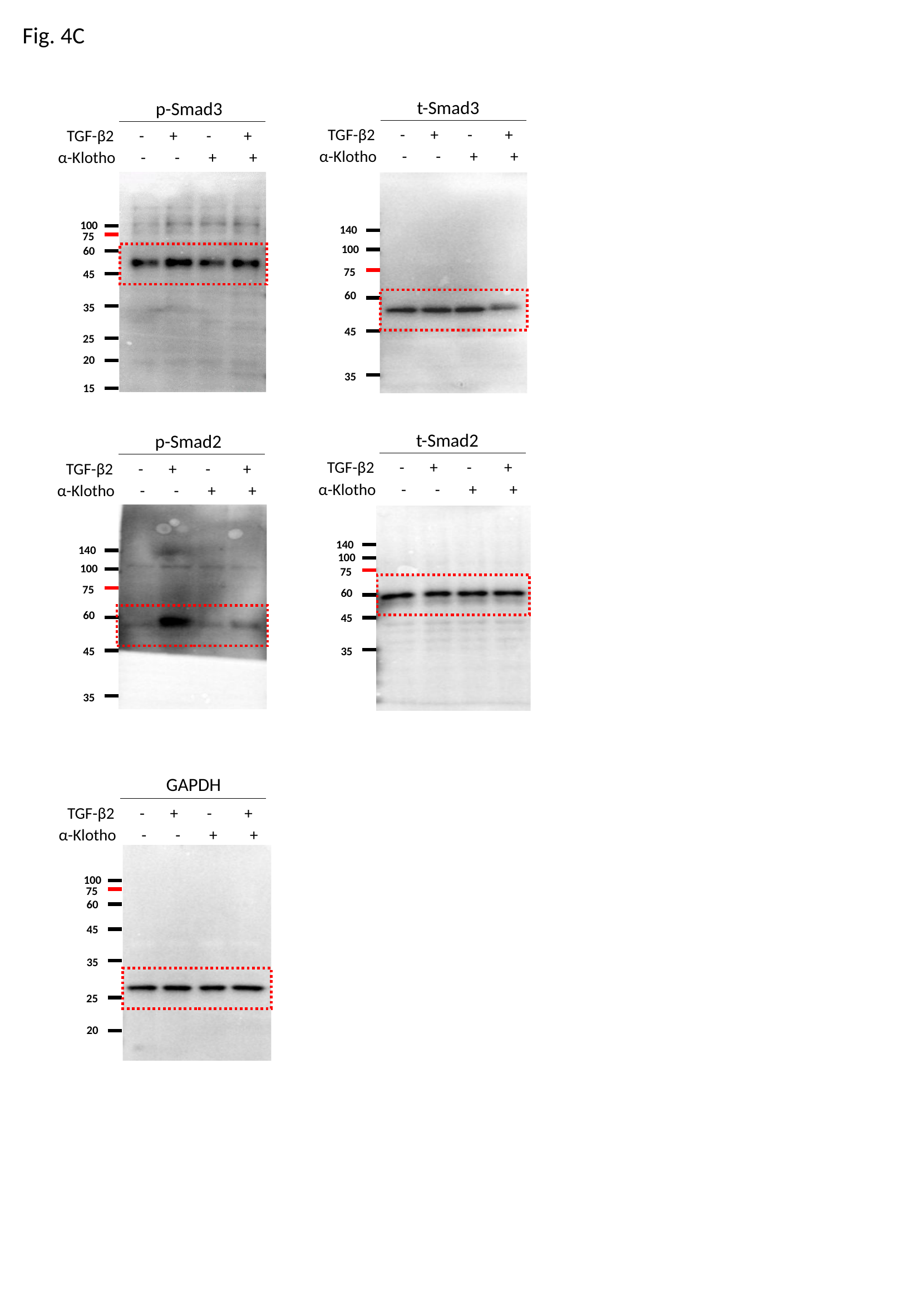

Fig. 4C
t-Smad3
p-Smad3
TGF-β2 - + - +
TGF-β2 - + - +
α-Klotho - - + +
α-Klotho - - + +
100
140
75
100
60
75
45
60
35
45
25
20
35
15
t-Smad2
p-Smad2
TGF-β2 - + - +
TGF-β2 - + - +
α-Klotho - - + +
α-Klotho - - + +
140
140
100
100
75
75
60
60
45
45
35
35
GAPDH
TGF-β2 - + - +
α-Klotho - - + +
100
75
60
45
35
25
20

## Slide 5
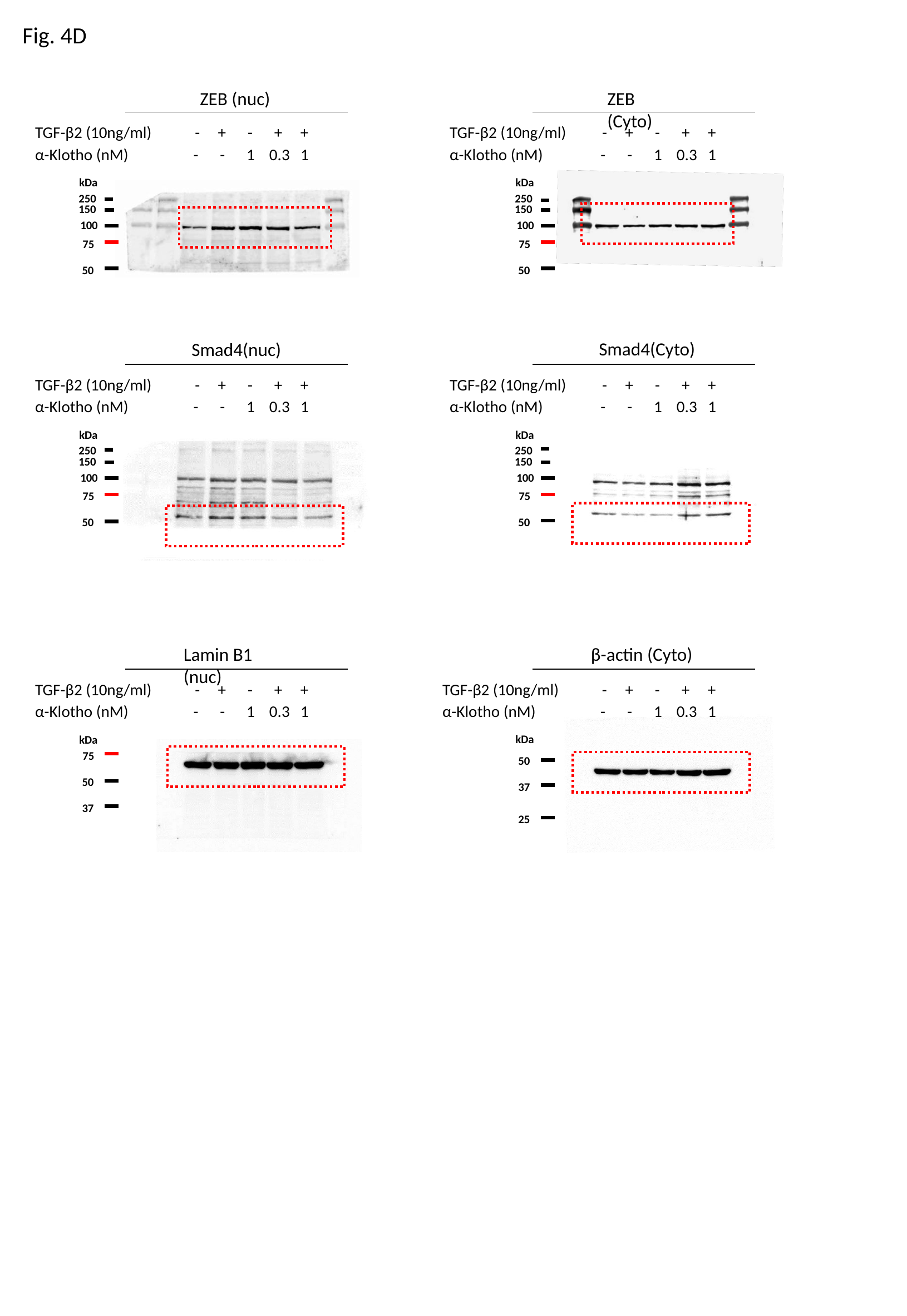

Fig. 4D
ZEB (nuc)
ZEB (Cyto)
TGF-β2 (10ng/ml) - + - + +
 TGF-β2 (10ng/ml) - + - + +
α-Klotho (nM) - - 1 0.3 1
 α-Klotho (nM) - - 1 0.3 1
kDa
kDa
250
250
150
150
100
100
75
75
50
50
Smad4(Cyto)
Smad4(nuc)
TGF-β2 (10ng/ml) - + - + +
 TGF-β2 (10ng/ml) - + - + +
α-Klotho (nM) - - 1 0.3 1
 α-Klotho (nM) - - 1 0.3 1
kDa
kDa
250
250
150
150
100
100
75
75
50
50
Lamin B1 (nuc)
β-actin (Cyto)
TGF-β2 (10ng/ml) - + - + +
TGF-β2 (10ng/ml) - + - + +
α-Klotho (nM) - - 1 0.3 1
α-Klotho (nM) - - 1 0.3 1
kDa
kDa
75
50
50
37
37
25

## Slide 6
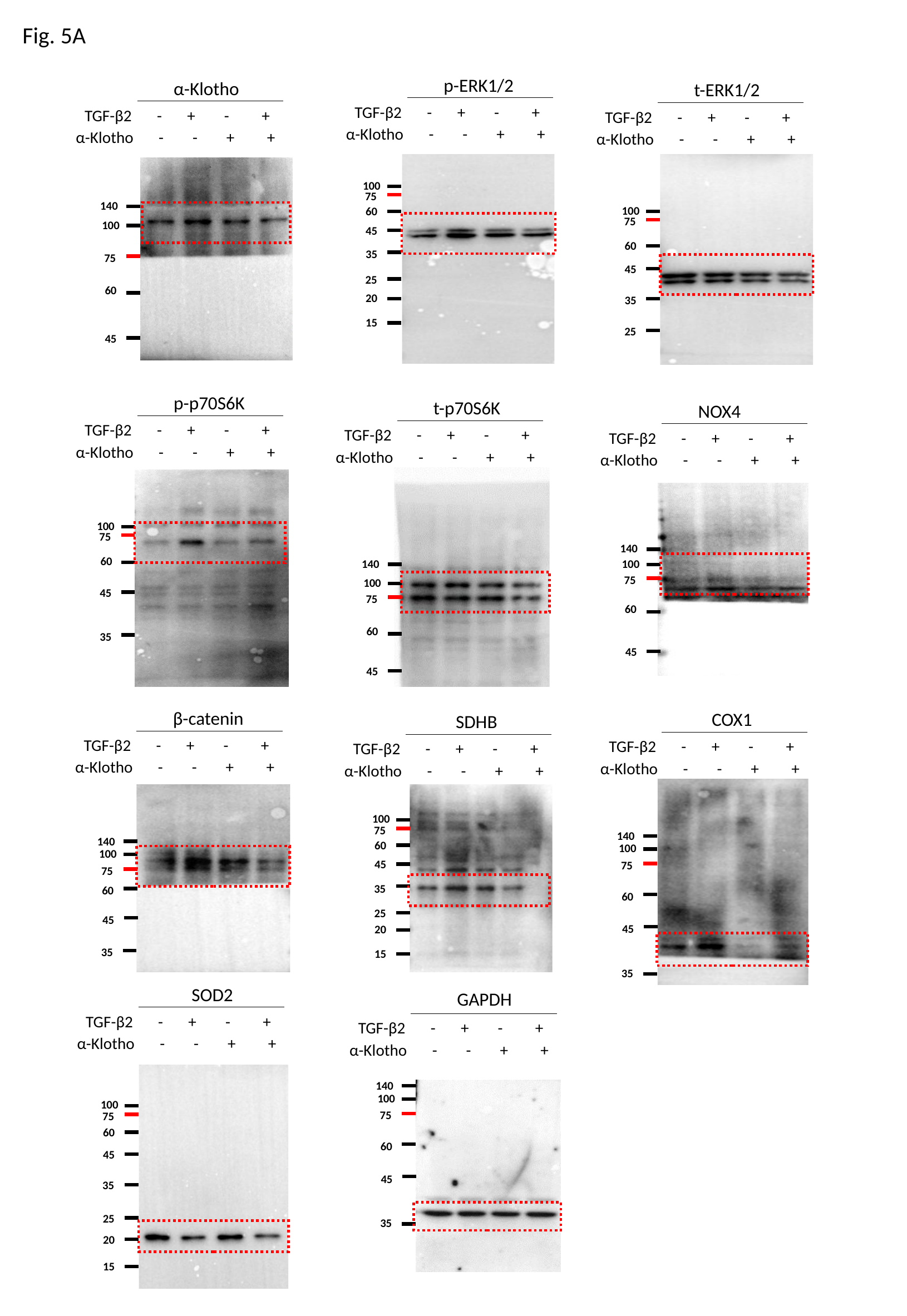

Fig. 5A
p-ERK1/2
α-Klotho
t-ERK1/2
TGF-β2 - + - +
TGF-β2 - + - +
TGF-β2 - + - +
α-Klotho - - + +
α-Klotho - - + +
α-Klotho - - + +
100
75
140
100
60
75
100
45
60
35
75
45
25
60
20
35
15
25
45
p-p70S6K
t-p70S6K
NOX4
TGF-β2 - + - +
TGF-β2 - + - +
TGF-β2 - + - +
α-Klotho - - + +
α-Klotho - - + +
α-Klotho - - + +
100
75
140
60
140
100
75
100
45
75
60
60
35
45
45
β-catenin
COX1
SDHB
TGF-β2 - + - +
TGF-β2 - + - +
TGF-β2 - + - +
α-Klotho - - + +
α-Klotho - - + +
α-Klotho - - + +
100
75
140
140
60
100
100
45
75
75
35
60
60
25
45
45
20
35
15
35
SOD2
GAPDH
TGF-β2 - + - +
TGF-β2 - + - +
α-Klotho - - + +
α-Klotho - - + +
140
100
100
75
75
60
60
45
45
35
25
35
20
15

## Slide 7
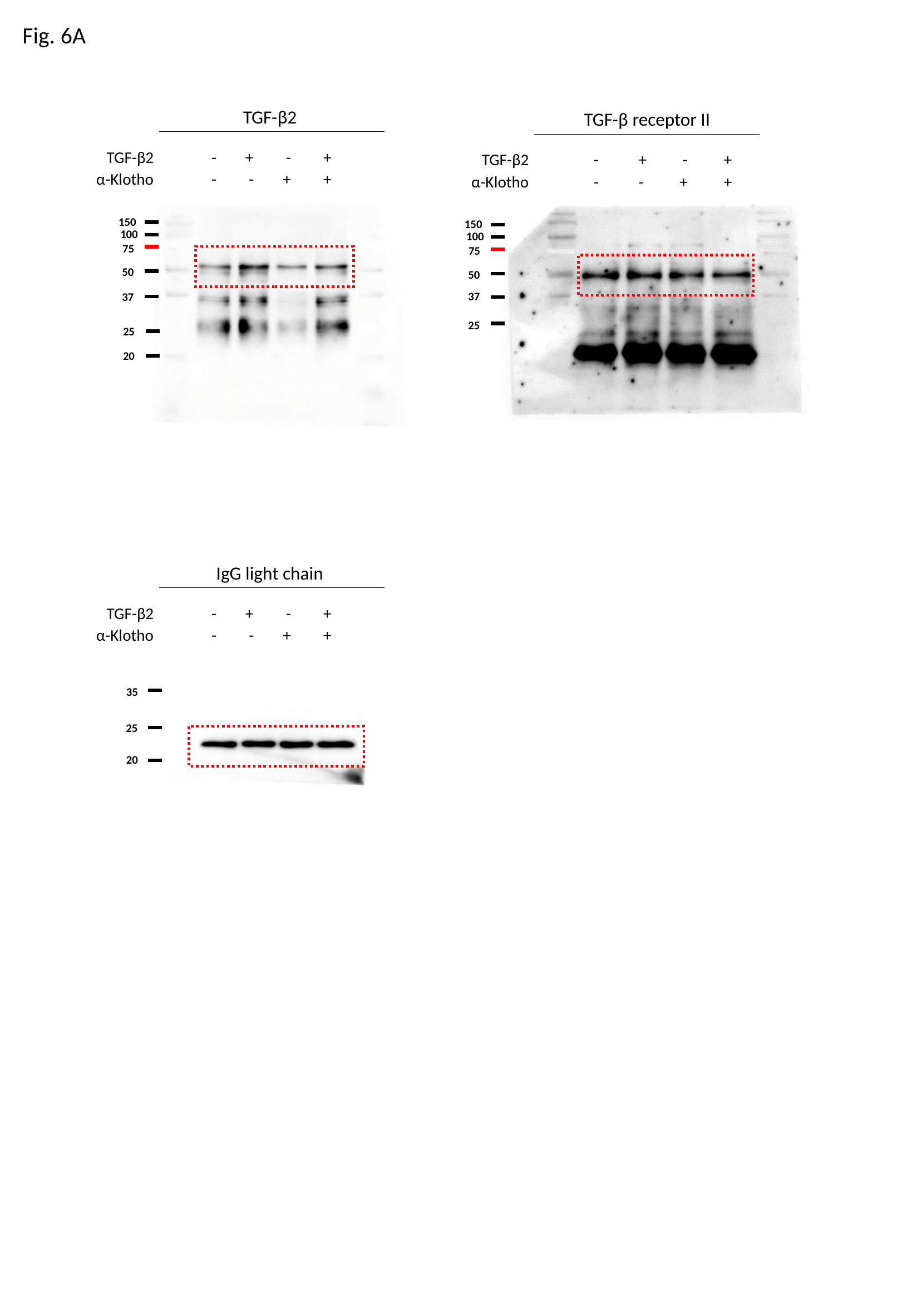

Fig. 6A
TGF-β2
TGF-β receptor II
TGF-β2 - + - +
TGF-β2 - + - +
α-Klotho - - + +
α-Klotho - - + +
150
150
100
100
75
75
50
50
37
37
25
25
20
IgG light chain
TGF-β2 - + - +
α-Klotho - - + +
35
25
20

## Slide 8
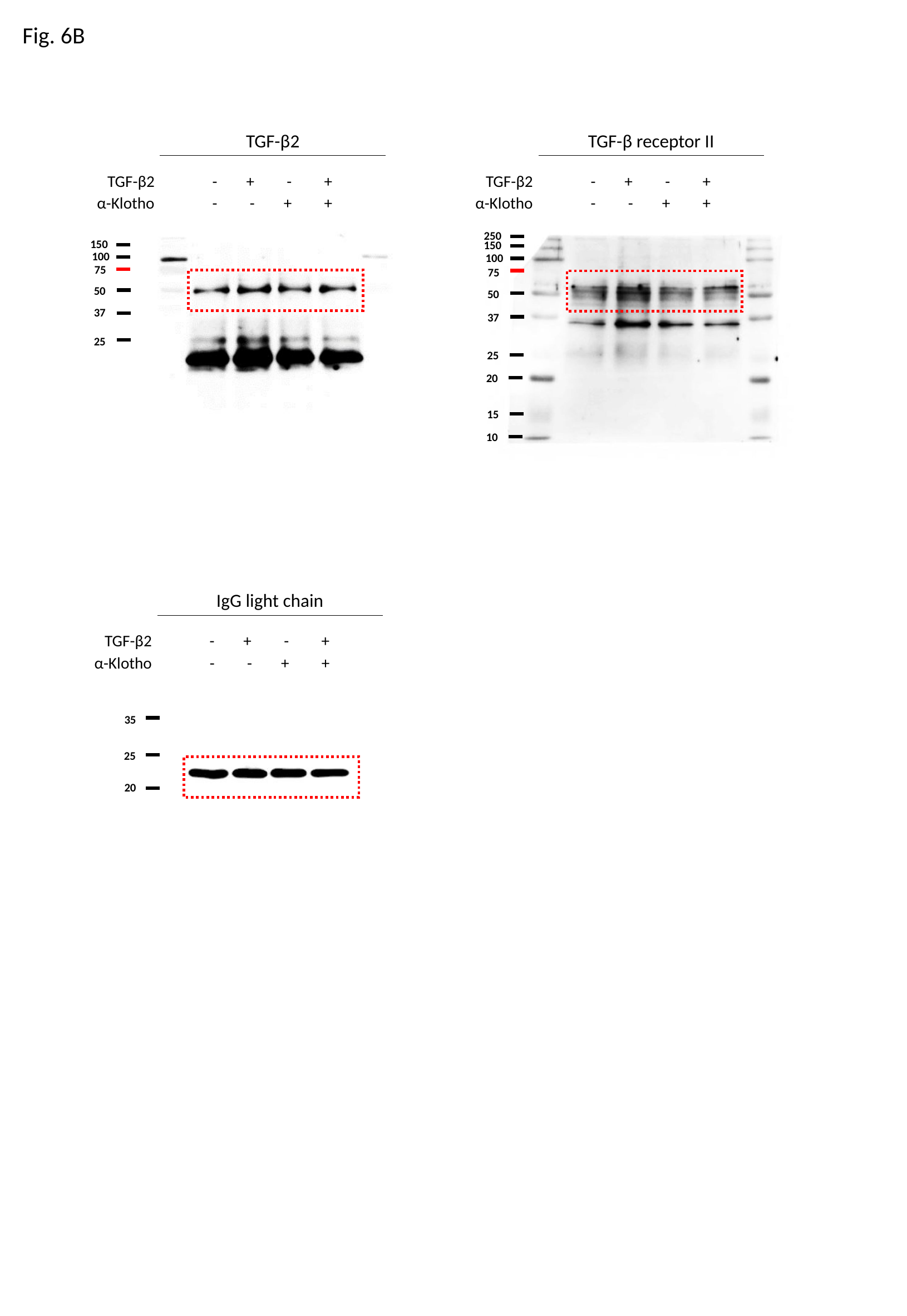

Fig. 6B
TGF-β2
TGF-β receptor II
TGF-β2 - + - +
TGF-β2 - + - +
α-Klotho - - + +
α-Klotho - - + +
250
150
150
100
100
75
75
50
50
37
37
25
25
20
15
10
IgG light chain
TGF-β2 - + - +
α-Klotho - - + +
35
25
20

## Slide 9
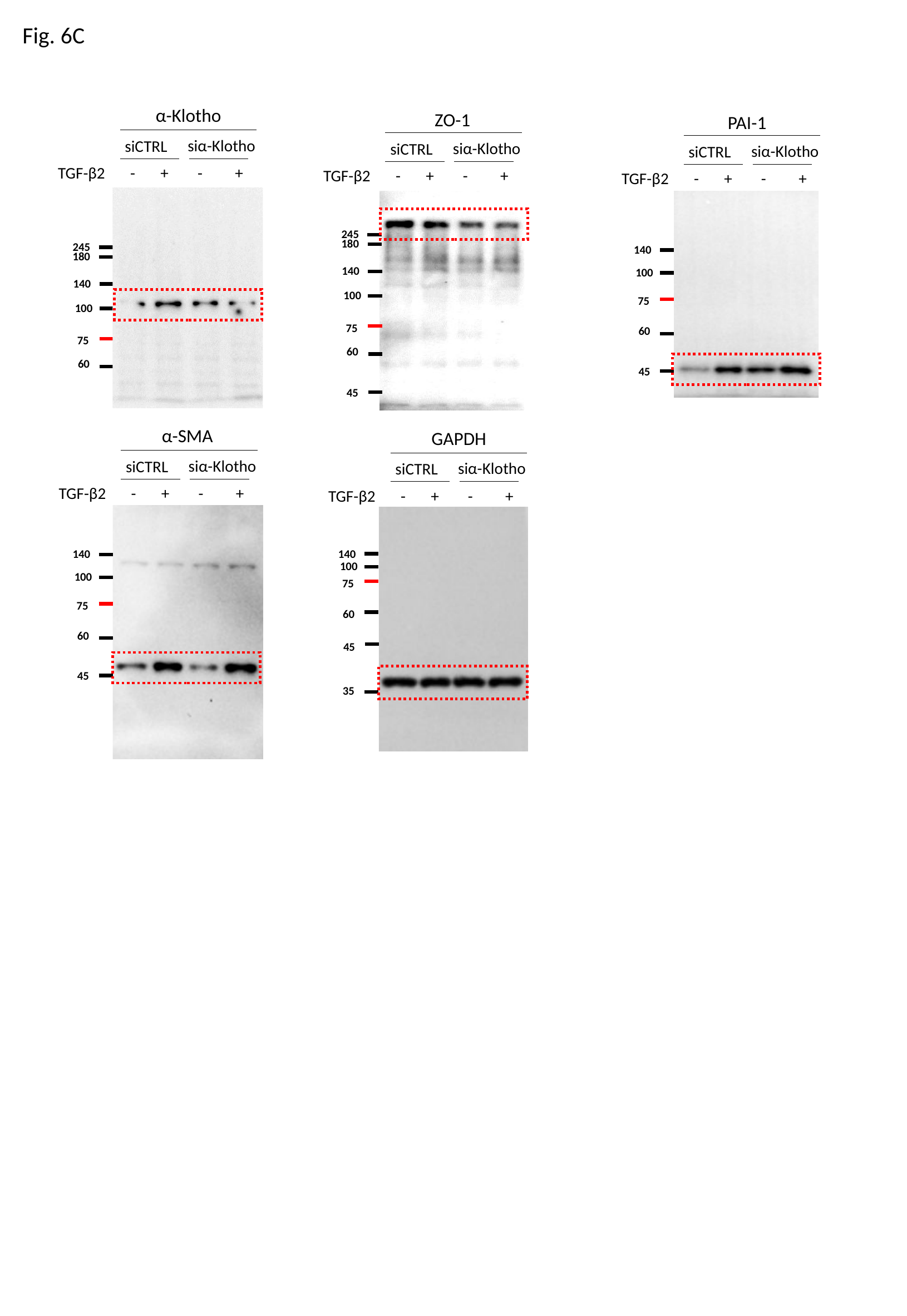

Fig. 6C
α-Klotho
ZO-1
PAI-1
siα-Klotho
siCTRL
siα-Klotho
siCTRL
siα-Klotho
siCTRL
TGF-β2 - + - +
TGF-β2 - + - +
TGF-β2 - + - +
245
180
245
140
180
140
100
140
100
75
100
75
60
75
60
60
45
45
α-SMA
GAPDH
siα-Klotho
siCTRL
siα-Klotho
siCTRL
TGF-β2 - + - +
TGF-β2 - + - +
140
140
100
100
75
75
60
60
45
45
35

## Slide 10
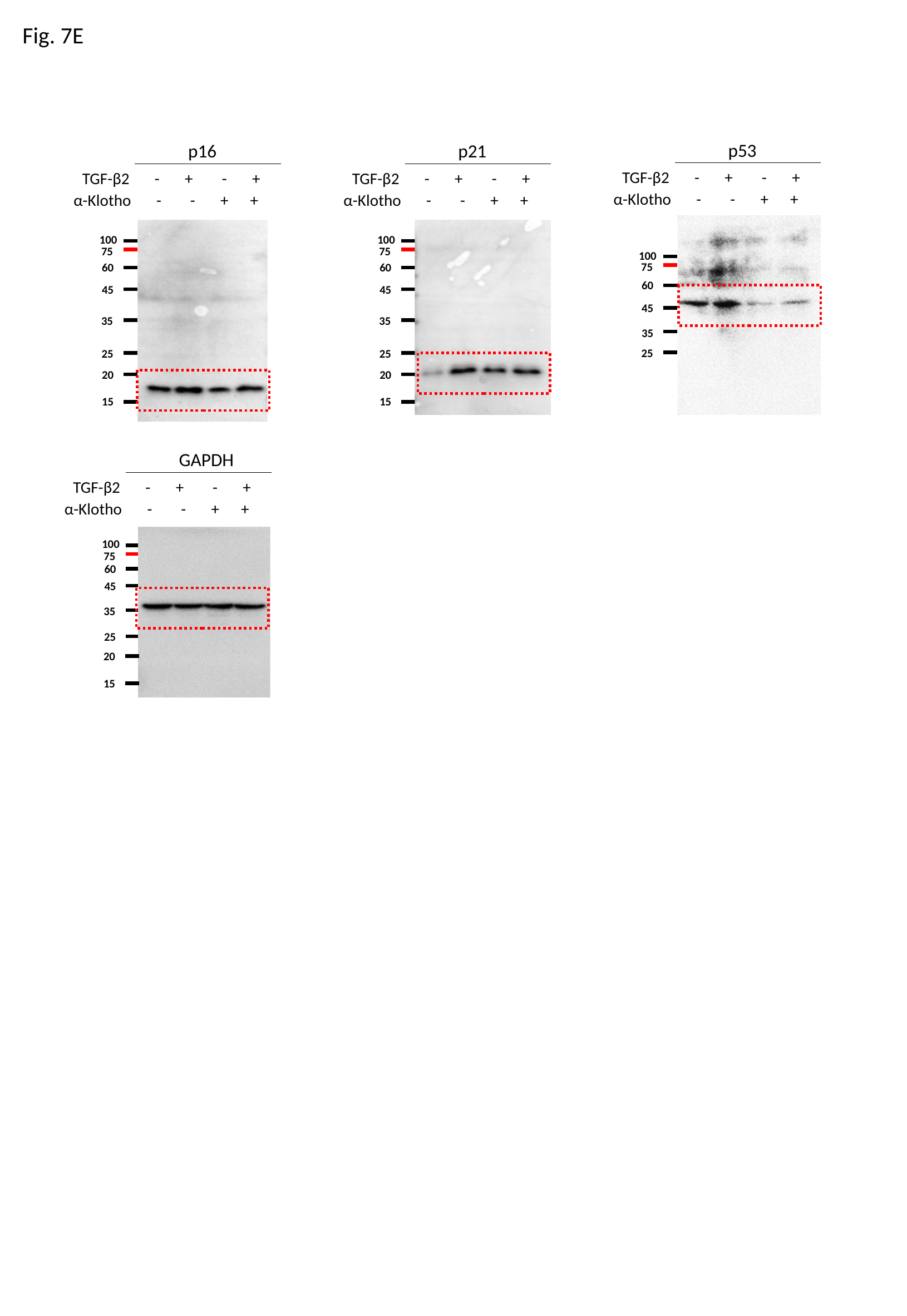

Fig. 7E
p53
p16
p21
TGF-β2 - + - +
TGF-β2 - + - +
TGF-β2 - + - +
α-Klotho - - + +
α-Klotho - - + +
α-Klotho - - + +
100
100
75
75
100
75
60
60
60
45
45
45
35
35
35
25
25
25
20
20
15
15
GAPDH
TGF-β2 - + - +
α-Klotho - - + +
100
75
60
45
35
25
20
15
